# A transposable element insertion in *AUX/IAA16* disrupts splicing and causes auxin resistance in *Bassia scoparia*

**DOI:** 10.1111/tpj.70339

**Published:** 2025-07-20

**Authors:** Jacob S. Montgomery, Neeta Soni, Sofia Marques Hill, Sarah Morran, Eric L. Patterson, Seth A. Edwards, Sandaruwan Ratnayake, Yu‐Hung Hung, Pratheek H. Pandesha, R. Keith Slotkin, Richard Napier, Franck Dayan, Todd Gaines

**Affiliations:** ^1^ Department of Agricultural Biology Colorado State University Fort Collins Colorado USA; ^2^ Department of Plant, Soil, and Microbial Sciences Michigan State University East Lansing Michigan USA; ^3^ Donald Danforth Plant Science Center St. Louis Missouri USA; ^4^ Division of Biological Sciences University of Missouri Columbia Missouri USA; ^5^ Division of Biology & Biomedical Sciences Washington University in St. Louis St. Louis Missouri USA; ^6^ School of Life Sciences University of Warwick Coventry UK

**Keywords:** herbicide resistance, retrotransposon, outlaw, linkage mapping, splice variant, kochia, surface plasmon resonance, degron, dicamba, 2,4‐D

## Abstract

A dicamba‐resistant population of kochia (*Bassia scoparia*) identified in Colorado, USA in 2012 was used to generate a synthetic mapping population that segregated for dicamba resistance. Linkage mapping associating dicamba injury with genotype derived from restriction‐site‐associated DNA sequencing identified a single locus in the kochia genome associated with resistance on chromosome 4. A mutant version of *Auxin/Indole‐3‐Acetic Acid 16* (*AUX/IAA16*; a gene previously implicated in dicamba resistance in kochia) was found near the middle of this locus in resistant plants. Long‐read sequencing of dicamba‐resistant plants identified a recently inserted long‐terminal repeat (LTR) retrotransposon TRIM element near the beginning of the second exon of *AUX/IAA16*, leading to disruption of normal splicing and a mutated degron domain. Stable transgenic lines of *Arabidopsis thaliana* ectopically expressing the mutant and wild‐type alleles of *AUX/IAA16* were developed. *Arabidopsis thaliana* plants expressing the mutant *AUX/IAA16* allele grew shorter roots on control media. However, transgenic root growth was less inhibited on media containing either dicamba (5 μM) or IAA (0.5 μM) when compared with non‐transgenic plants or those expressing the wild‐type allele of *AUX/IAA16. In vitro* assays indicate reduced binding affinity and more rapid dissociation of the mutant AUX/IAA16 with TIR1 in the presence of several auxins, and protein modeling suggests the substitution of the glycine residue in the degron domain of AUX/IAA16 is especially important for resistance. A fitness cost associated with the mutant allele of *AUX/IAA16* has implications for resistance evolution and management of kochia populations with this resistance mechanism.

## Significance Statement

Auxin mimics are among the most important and widely used herbicides in modern agriculture. Evolution of weeds that are resistant to these herbicides threatens sustainable crop production. Understanding the basis of auxin herbicide resistance improves our understanding of rapid evolution and informs the development of improved weed control technologies. Additionally, auxin‐resistance mutations and their pleiotropic effects help us understand auxin perception and signaling. We describe a transposable element insertion within an herbicide target site gene that alters transcript splicing and reduces synthetic and natural auxin perception.

## INTRODUCTION

Weed control in modern agricultural production systems relies largely on herbicides. The first synthetic herbicides to be commercialized (e.g., 2,4‐D; described by Quastel, 1950) were eventually determined to mimic the endogenous plant hormone auxin. The low cost of these synthetic auxin herbicides, matched with their ability to selectively control broadleaf weeds in cereal production, led to the rapid adoption of herbicide‐based weed management. As new herbicides were discovered and commercialized, mixing multiple herbicides in a single spray mixture has allowed for robust control of many of the most important weeds in diverse agricultural settings. While the evolution of herbicide‐resistant weed populations has reduced the efficacy of many herbicides, the evolution of resistance to synthetic auxin herbicides has been slower and less frequent than for other modes of action (Busi et al., 2018; Heap, 2024). Thus, some of the oldest herbicides remain some of the most widely used across many agricultural settings. In fact, auxinic herbicide resistance traits were engineered into dicot crops (e.g., soybean and cotton) to provide farmers more control options against weed populations that had evolved resistance to herbicides with different modes of action unrelated to auxin signaling, such as glyphosate (Behrens et al., 2007; Wright et al., 2010). These traits expanded the potential use and value of two of the most used synthetic auxin herbicides, dicamba and 2,4‐D. This expansion has had the unintended effect of increasing selection pressure for resistance alleles and is correlated with a recent increase in the number of resistance reports to these herbicides. Beyond weed control, these synthetic auxin compounds have been crucial to experiments that aim to understand auxin perception and signaling, perhaps the most complex of all plant hormone signaling pathways (Ma et al., 2017).

While the physiological mechanism of action for many herbicides has been well understood for decades, the cascade of cellular responses following the application of a synthetic auxin herbicide is convoluted and is the subject of many recent studies. The topic has been reviewed several times, with new experimental results incrementally advancing our knowledge of the key factors leading to plant death (Christoffoleti et al., 2015; Grossmann, 2010; McCauley et al., 2020; Song, 2014). Auxin, whether synthetic or natural, first binds to one or more of the isoforms of its receptor protein, Transport Inhibitor Response 1/Auxin‐Signaling F‐Box (TIR1/AFB) (Dharmasiri et al., 2005; Kepinski & Leyser, 2005). Once bound to TIR1/AFB, auxin acts as a ‘molecular glue’, mediating the interaction between TIR1/AFB and co‐receptor Auxin/Indole‐3‐Acetic Acid (AUX/IAA) proteins. Identification of conserved domains and experimentation have produced a list of residues in TIR1/AFB and AUX/IAA that are vital for this interaction (Ramans‐Harborough et al., 2023; Tan et al., 2007; Uzunova et al., 2016). This interaction leads to the ubiquitination and rapid degradation of AUX/IAA proteins (Gray et al., 2001; Zenser et al., 2001). Because AUX/IAA proteins act as transcriptional regulators by inhibiting auxin response factors (ARFs), which are transcription factors that promote the expression of auxin‐responsive genes, AUX/IAA degradation leads to the modulation of auxin‐responsive gene expression (Grossmann, 2010). *TIR1/AFB* and *AUX/IAA* are each multigene families with varying numbers of homologs. The diversity in gene members of these families allows for many possible combinations of TIR1/AFB and AUX/IAA, which in turn allow for tissue, temporal, and environmental regulation of auxin responses. Different synthetic auxin herbicides likely promote the interaction of certain AUX/IAA proteins with certain TIR1/AFB proteins more efficiently, but this network of interactions is still not well understood. For example, LeClere et al. (2018) show that AUX/IAA16 preferentially binds with TIR1 and AFB6 (a homolog of AFB found in members of Amaranthaceae, but not Brassicaceae) in the presence of some auxin analogs, but not others.

A disruption in any step of this auxin signaling system (e.g., TIR1/AFB binding auxin, AUX/IAA interacting with TIR1/AFB, or the ubiquitination and degradation pathway of AUX/IAA) could cause reduced activity of synthetic auxin herbicides (LeClere et al., 2018; Ruegger et al., 1998; Todd et al., 2020). Thus, evolution of synthetic auxin resistance should be relatively simple and common across weed species in the face of incredibly pervasive and strong selection pressure. However, mutations causing resistance to synthetic auxin herbicides are likely also to cause a reduction in fitness compared with the wild‐type due to the conserved and vital function of these proteins (Roux & Reboud, 2005; Wu et al., 2021), suggesting that resistance mutations are likely to be purged in the absence of synthetic auxin herbicide selection pressure during rotations in land and herbicide use. Nevertheless, there are several reported cases of synthetic auxin herbicide resistance becoming established in some weed species. Interestingly, in cases in which the genetic basis of resistance has been determined, all reports pertain to mutations in or near the degron domain of *AUX/IAA* genes. LeClere et al. (2018) and Ghanizadeh et al. (2024) report, in closely related species, different mutations of a specific glycine residue in *AUX/IAA16* that is known to be vital to normal auxin signaling. In unrelated species, de Figueiredo, Barnes, et al. (2022) report a 27‐bp deletion that shortens the distance between the degron and PB1 domains of AUX/IAA2, and Krishnan et al. (2024) report a double deletion mutation flanking the degron region of *AUX/IAA20*.

Here, we seek to understand the genetic and molecular basis of dicamba resistance in a population of kochia (*Bassia scoparia*) collected from Colorado, USA. We use linkage mapping to identify a region of the genome that segregates with resistance and contains a mutant version of *AUX/IAA16*. Long read sequencing allowed us to identify a transposable element insertion in the coding region of *AUX/IAA16* that disrupts normal splicing and changes several amino acids in the protein product relative to the wild‐type. Ectopic expression in *Arabidopsis thaliana* proves this mutant *AUX/IAA16* is sufficient to cause resistance to dicamba. Surface plasmon resonance assays detect a reduction in binding affinity and more rapid dissociation for the mutant AUX/IAA protein in the presence of several auxin analogs, and protein modeling points to the importance of a glycine‐to‐threonine substitution in the degron domain of this mutant. This mutation also seems to confer a fitness cost associated with this mechanism of dicamba resistance, with implications for agricultural management of dicamba‐resistant weed populations.

## RESULTS AND DISCUSSION

### Characterization of herbicide resistance

**Figure 1:**
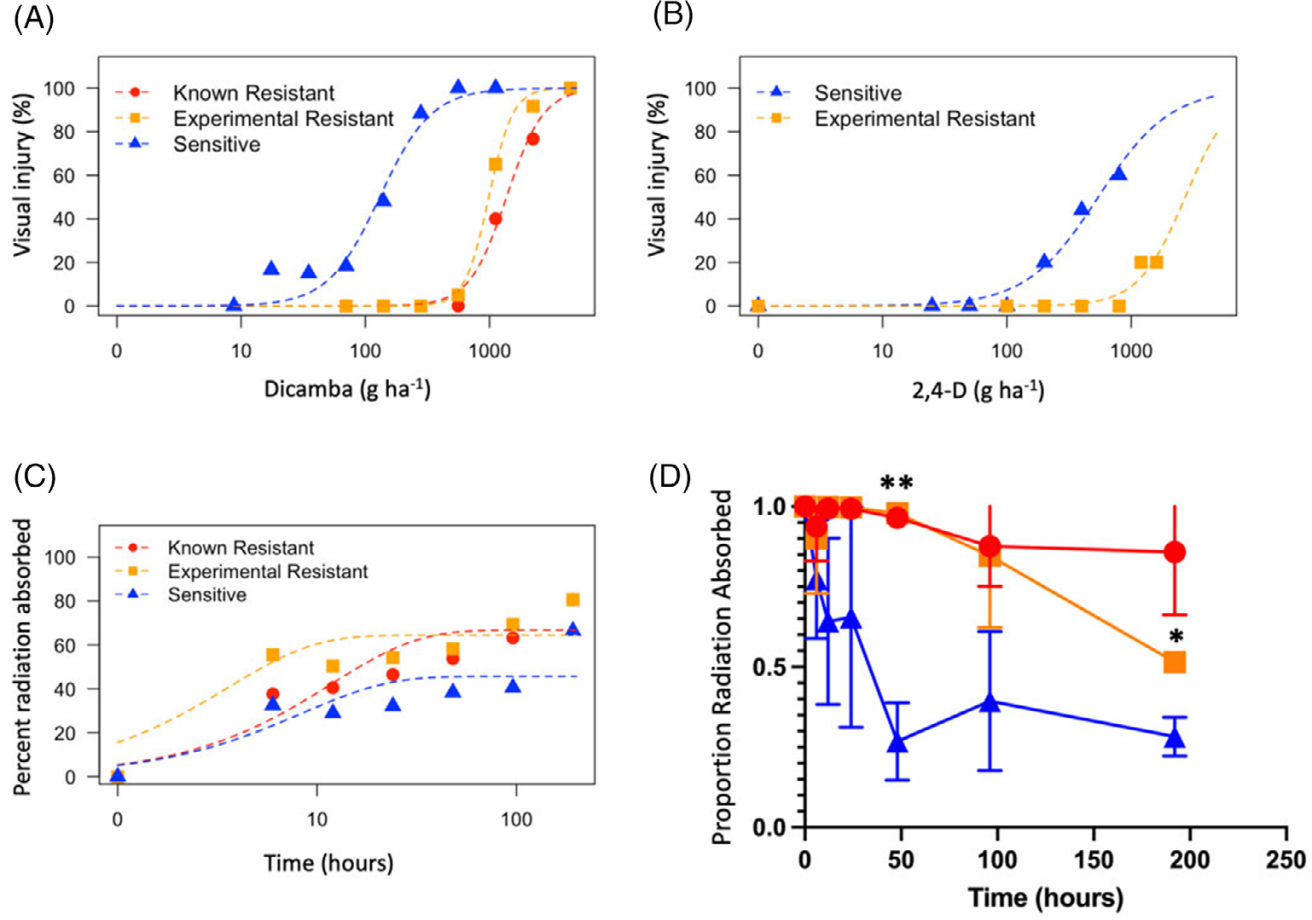
Characterization of auxin herbicide resistance in populations of *Bassia scoparia*. Dose–response curves showing magnitude of resistance toward dicamba (A) and 2,4‐D (B) for the M32 population of *Bassia scoparia* (experimental resistant) compared with a known dicamba‐sensitive population and known dicamba‐resistant population (LeClere et al., 2018; Pettinga et al., 2018). (C) Time course of ^14^C dicamba absorption showing lack of herbicide absorption is not associated with dicamba resistance in dicamba‐resistant populations of *B. scoparia*. (D) Proportion of absorbed ^14^C dicamba retained in the treated leaf showing less dicamba exits the treated leaf in dicamba‐resistant populations compared with a dicamba‐sensitive population. Error bars represent standard error. *P < 0.05, **P < 0.01. Populations are represented by the same colors and symbols as in (A–C).

Dose–response experiments were conducted to confirm and quantify the level of dicamba and 2,4‐D resistance in a population of kochia (named M32) collected from Akron, Colorado, USA (Figure 1A,B). Parameter estimates for two‐parameter log‐logistic models for each population used in these experiments are available in Tables S1 and S2. In the dicamba experiment, populations M32 and 9425 (a known dicamba‐resistant kochia population previously characterized by LeClere et al. (2018) and Pettinga et al. (2018)) had ED_50_ estimates 7.6 (*P* < 0.0001) and 10.5 (*P* < 0.0001) times greater than an herbicide‐sensitive kochia population (named 7710; described by Preston et al. (2009)). The ED_50_ parameter represents the dose of herbicide needed to cause 50% injury to that specific population. These estimates were significantly different from the herbicide‐sensitive control and from each other (*P* < 0.0001), indicating dicamba resistance in M32 is not as high as in 9425. Nevertheless, the ED_50_ estimate for both resistant populations was greater than the recommended field rate of dicamba (560 g ha^−1^), indicating neither population would likely be controlled by dicamba in a field setting. Similarly, in the 2,4‐D experiment, the ED_50_ estimate for M32 was 5.0 (*P* < 0.0001) times greater than that of the herbicide‐sensitive control and greater than the recommended field rate for 2,4‐D (approximately 560 g ha^−1^). It is worth noting that the ED_50_ estimate for the herbicide‐sensitive population (543 g ha^−1^) is nearly equal to the recommended field rate for 2,4‐D application. Thus, 2,4‐D is not likely to provide good control of kochia in general.

While herbicide‐sensitive plants show epinasty starting approximately 5 days after treatment (DAT) with dicamba or 2,4‐D, resistant plants in our study from an inbred population do not have any symptoms when treated with a field rate of either herbicide. This lack of initial injury suggests that resistant plants either rapidly metabolize/sequester the herbicides before they cause injury (Behrens et al., 2007; Ge et al., 2010) or possess mutant target site(s) that reduce their effect on their molecular target(s) (de Figueiredo, Küpper, et al., 2022; LeClere et al., 2018; Walsh et al., 2006). Highly efficient resistance has been engineered into some crops using genes coding for metabolic enzymes from bacteria (Behrens et al., 2007; Wright et al., 2010). In these cases, no injury symptoms are observed at field rates. However, in documented cases of metabolic auxin herbicide resistance, resistant plants often do show symptoms of herbicide damage but eventually recover (de Figueiredo, Barnes, et al., 2022; Todd et al., 2024).

To understand if physiological mechanisms prevent dicamba from being absorbed or translocated within resistant plants, radiolabeled dicamba was used to track its uptake and movement in dicamba‐resistant and ‐sensitive plants. Significantly less dicamba was absorbed by herbicide‐sensitive plants over 192 h compared with the dicamba‐resistant M32 and 9425 populations (*P* = 0.036 and *P* = 0.019 respectively), but the rate of absorption (time required for 90% absorption) was not significantly different between populations (Figure 1C; Table S3). Therefore, reduced dicamba absorption is not responsible for resistance. Time series data for the translocation of radiolabeled dicamba out of the treated leaf did not fit well to any tested models, so *t*‐tests were used to determine if means differed between resistant and sensitive populations at each time point (Figure 1D). Dicamba‐resistant plants translocated less dicamba out of the treated leaf compared with the sensitive plants. These results show similar absorption and translocation profiles between M32 and 9425 populations, suggesting the M32 population may use a resistance mechanism similar to the 9425 population, where resistance is caused by a mutation in *AUX/IAA16* (LeClere et al., 2018).

### A single locus on chromosome 4 is associated with dicamba resistance

**Figure 2:**
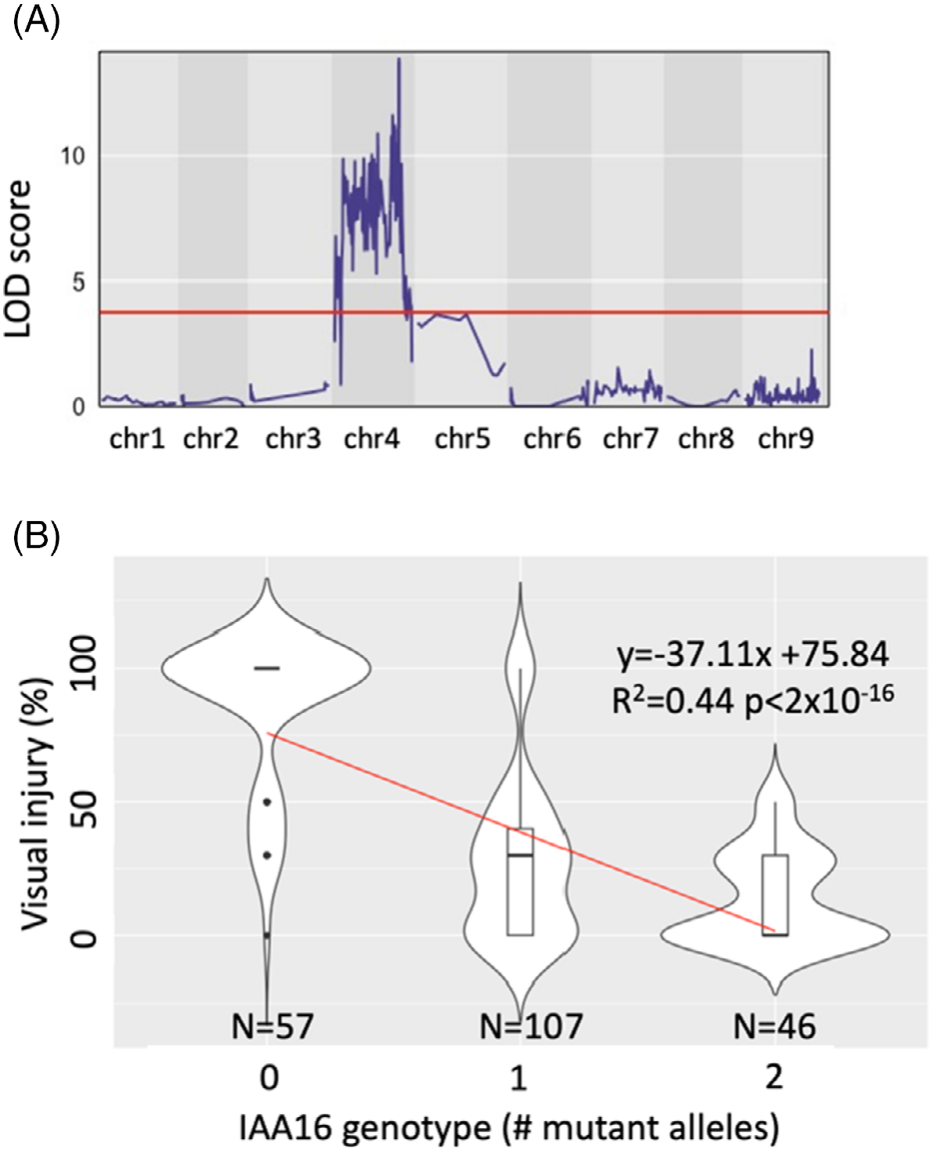
Genetic mapping of herbicide resistance loci in Bassia scoparia. (A) Quantitative trait loci (QTL) genome scan from a segregating F_3_ population derived from a biparental cross of herbicide‐resistant and ‐sensitive *Bassia scoparia* plants. Red line represents a significance threshold determined by Bonferroni‐adjustment of alpha = 0.05 followed by conversion to LOD as described by Nyholt (2000). (B) Violin plot displaying the effect of *AUX/IAA16* genotype on visual injury 21 days after treatment with dicamba in an independent segregating F_3_ population of *B. scoparia*. Red line represents a linear model fitted to all points with equation, adjusted *R* ^2^, and *P*‐value of an *F*‐test testing the effect of genotype listed at the top right. Number of observations for each genotype listed at the base of the graph.

A quantitative trait loci (QTL) mapping approach was taken to identify regions of the genome associated with dicamba resistance. Segregating F_3_ populations were developed from multiple independent biparental crosses using a dicamba‐resistant plant as the male parent and a dicamba‐sensitive plant as the female parent. We expected to find a single dominant locus controlling dicamba resistance because the ratio of alive: dead F_3_ plants following dicamba treatment at a rate of 560 g ha^−1^ (200:85) did not significantly differ from 3:1 (chi‐square test; *P* = 0.06). A double‐digest restriction site‐associated DNA sequencing (ddRADseq) protocol was used to determine genotypes of 103 segregating F_3_ plants at 1592 variant loci across the genome. A genome scan identified a large section of chromosome 4 that is associated with reduced visual injury following dicamba treatment. This is the first linkage mapping experiment in this species, so it is unclear how frequent recombination is and if strong linkage is responsible for such a broad peak. Alternatively, multiple loci along chromosome 4 may be contributing to resistance. In any case, a peak was determined for this QTL that ranges from 87.64837 to 87.99697 Mbp on chromosome 4 (Figure 2A). This interval contains 23 gene models, none of which are *AUX/IAA* genes, and is very near to Bs.00g107500 (*AUX/IAA16*) at 87.38 Mbp. This gene is important for auxin perception and has been implicated in auxin herbicide resistance in kochia and a closely related species (Ghanizadeh et al., 2024; LeClere et al., 2018). While two other *AUX/IAA* genes (Bs.00g107340; *AUX/IAA10* found at 87.18 Mbp and Bs.00g107550; *AUX/IAA22* found at 87.44 Mbp) are also found near this region, transcript sequencing did not reveal non‐synonymous changes near the degron domain. In plants from an independent segregating F_3_ line, the genotype of *AUX/IAA16* explained 43% of variation in visual injury following dicamba treatment at 560 g ha^−1^ (Figure 2B). While survival at this rate of dicamba may be dominant, visual injury collected from the same plants is incompletely recessive (Figure 2B) with a calculated degree of dominance of −0.58 (Falconer, 1964). This means that plants heterozygous for this mutation generally survived but showed more injury than homozygous mutant plants. This dominance for the survival phenotype has important implications for the spread of herbicide resistance alleles. For instance, a dominant trait is likely to spread more quickly throughout a population, but is less likely to be driven to fixation under selection (Holmes et al., 2022).

### A transposable element insertion in *AUX/IAA16* causes a change in exon splicing

**Figure 3:**
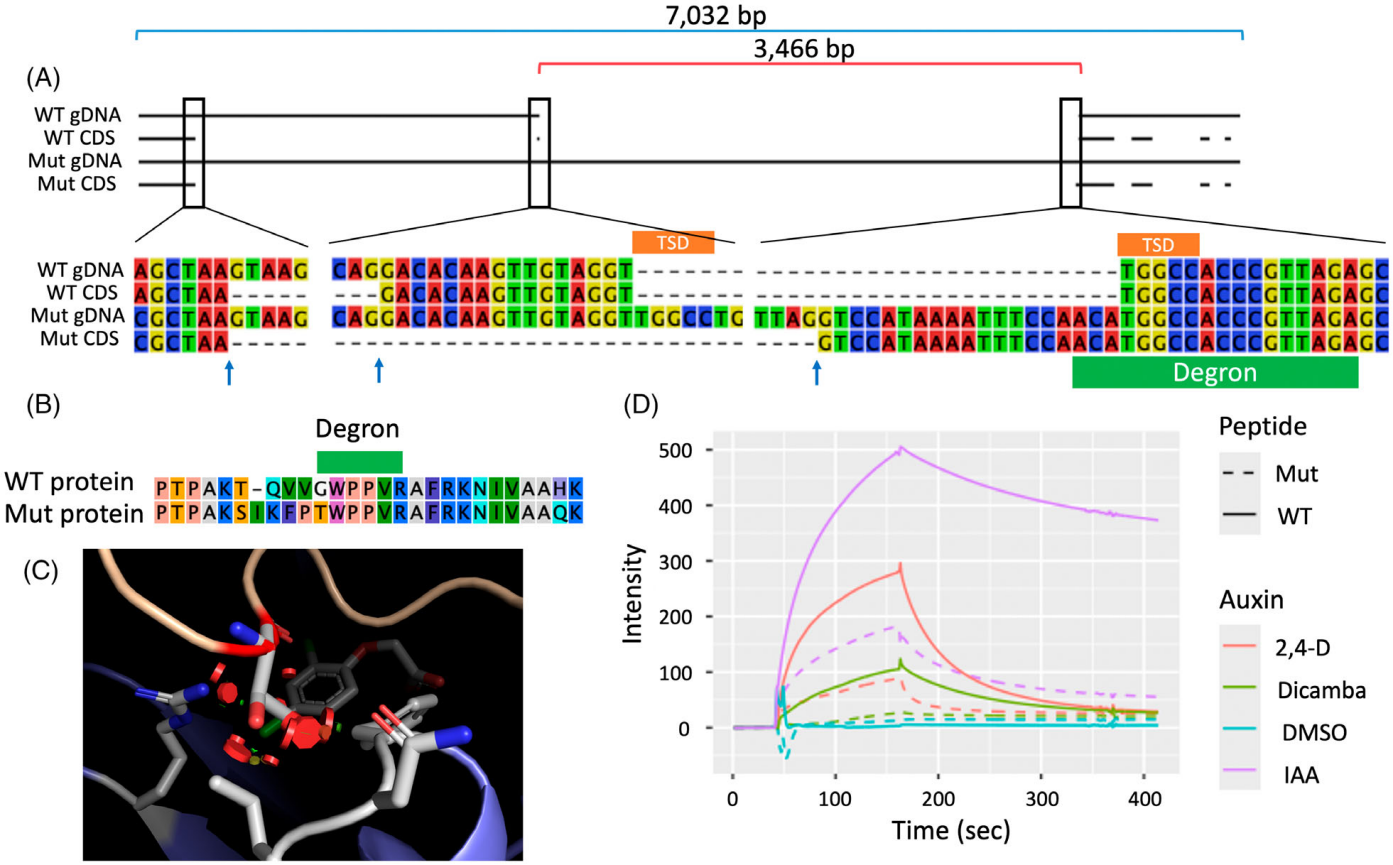
Characterization of a transposable element insertion in *AUX/IAA16*_*Mut*_ and its effect on protein structure and function. (A) Genomic and coding sequence for *AUX/IAA16* alleles detected in a mapping population segregating for dicamba resistance. Base pair positions are indicated above along with total length of the mutant gene (blue) and transposable element insertion (red). Splice sites around canonical splice motifs are indicated by blue arrows, target site duplications by orange boxes, and codons that encode the degron domain by a green box. (B) Amino acid sequence of mutant (Mut) and wild‐type (WT) isoforms of AUX/IAA16 around the degron domain. Amino acid positions are indicated above the sequences, and all amino acids not shown are conserved between alleles. (C) Crystal structure of IAA7 (tan) bound to TIR1 (blue) showing the glycine 127 residue (red) superimposed over the predicted structure if this residue is substituted for threonine, colored by element (carbon = gray, oxygen = red, nitrogen = blue, chlorine = green). Residues of TIR1 that are within 4 Å of the threonine residue are colored by element, and 2,4‐D is in the background, colored by element. Steric clashes are shown as red discs. (D) Binding and dissociation of AUX/IAA16 peptides with AtTIR1 in the presence of various auxins as determined by surface plasmon resonance experiments.

Amplification of *AUX/IAA16* transcripts from cDNA of kochia plants homozygous for either parental allele of the QTL discovered on chromosome 4 produced only one amplicon size for each genotype. While this suggests the production of one splice variant in each genotype, the use of primers at the beginning and end of the gene only allows us to identify transcripts that include these regions. Thus, we may be missing truncated forms of the transcript that do not include the primer binding site near the end of the canonical gene. Sanger sequencing of *AUX/IAA16* transcripts from these plants revealed near identical translated protein sequences between the parental genotypes except for the region around the degron domain (Figure 3B; Table S4). The gene model for *AUX/IAA16* predicts five exons in wild‐type plants, with the degron of *AUX/IAA16* located near the splice junction of exons one and two (Figure 3A). To understand the sequence of genomic DNA in and around *AUX/IAA16*, a plant homozygous for the resistant parent allele of *AUX/IAA16* was used for PacBio HiFi sequencing. After *de novo* assembly, we discovered a 3466 bp insertion at the beginning of exon two of *AUX/IAA16* (Figure 3A). The insertion disrupts splicing of *AUX/IAA16*, replacing 16 base pairs at the beginning of exon, two in the wild‐type with the final 19 bases of the insertion in the mutant (Figure 3A). The mutant allele will herein be referred to as *AUX/IAA16*_*Mut*_, while the wild‐type allele will be referred to as *AUX/IAA16*_*WT*_. This insertion results in the addition of one amino acid and the replacement of five more in the final protein product (Figure 3B; Table S4). The new splice sites flank canonical GT and AG motifs. This change includes a substitution of a glycine to threonine at the N‐terminal end of the degron domain (Figure 3B; Table S4). LeClere et al. (2018) showed that a substitution of this glycine to asparagine in AUX/IAA16 is sufficient for dicamba resistance in kochia. Similarly, Ghanizadeh et al. (2024) associate a substitution of this glycine to aspartic acid with dicamba resistance in *Chenopodium album*, a closely related species. Nuclear magnetic resonance experiments by Ramans‐Harborough et al. (2023) show that this glycine is especially involved in the interaction between AUX/IAA and TIR1, and our protein modeling shows that the larger side group of threonine contacts the surface of the TIR1 binding pocket and causes steric hindrance of the IAA/TIR1 interaction (Figure 3C). The rest of the mutant residues do not interact with the binding pocket of TIR1 and are within a disordered domain of AUX/IAA; thus, they are unlikely to affect IAA/TIR1 interactions (Ramans‐Harborough et al., 2023; Tan et al., 2007). Given the demonstrated importance of this specific glycine residue, we hypothesize that the glycine to threonine substitution is the major driver of dicamba resistance. However, these mutations are unlikely to affect the ability of AUX/IAA16 to interact with ARF proteins, given that there are no mutations that affect protein sequence of the PB1 domain, which participates in the protein–protein interaction between AUX/IAA and ARF proteins (Table S4; Korasick et al., 2014).

**Figure 4:**
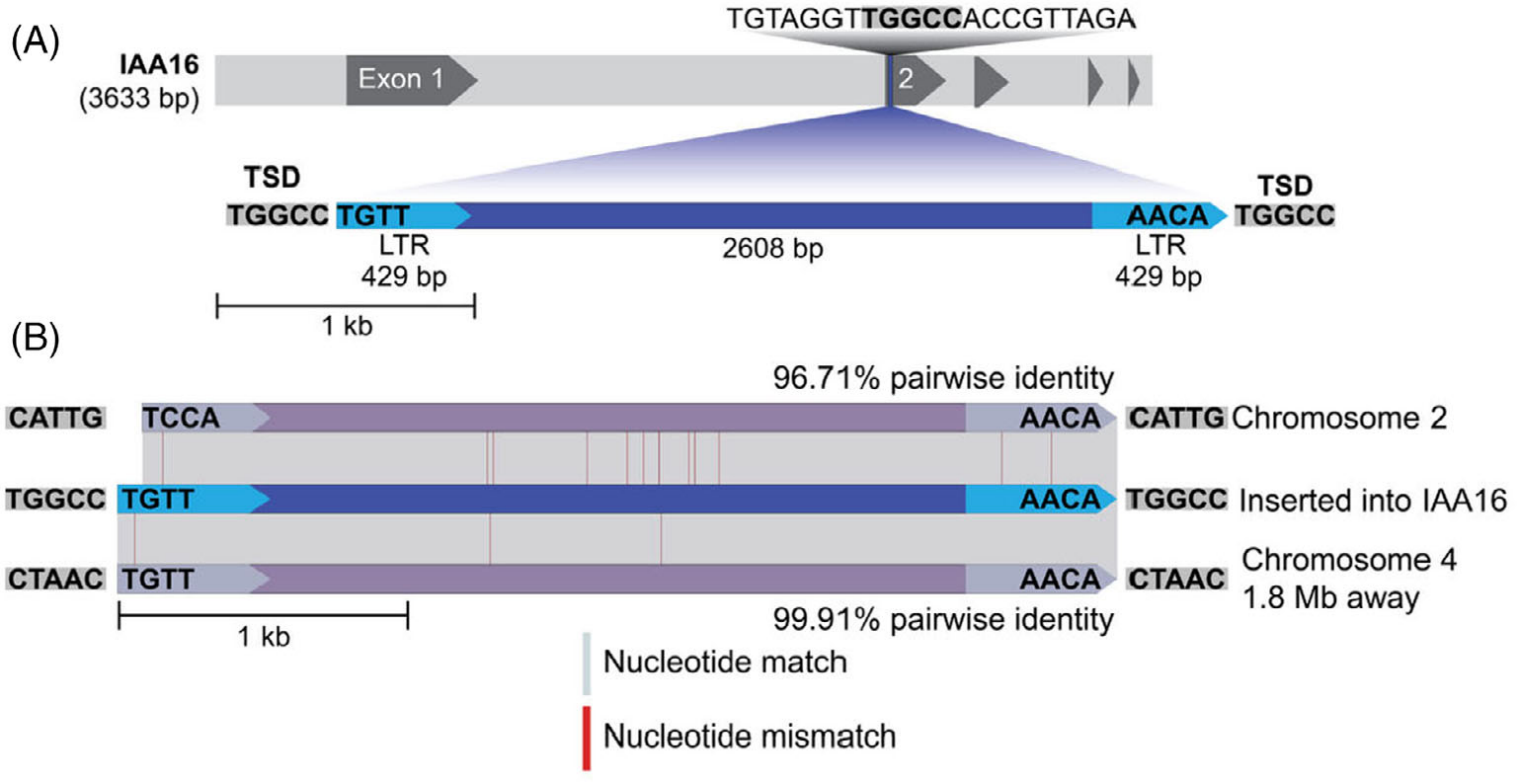
Characterization of non‐autonomous LTR retrotransposon, *Outlaw*, that was inserted in *AUX/IAA16*_*Mut*_. (A) *AUX/IAA16* gene structure with 3466 bp *Outlaw* insertion and the target site duplications (TSDs) shown in gray boxes. The terminal 4 nucloetides of the LTR on the edges of *Outlaw* are a conserved feature of TRIM TEs and are shown inside their respective LTRs. (B) Alignment of highly similar TRIM elements from the *Bassia scoparia* genome similar to *Outlaw* that inserted into *AUX/IAA16*_*Mut*_. Annotations and color code are the same from part (A).

The 3466 bp insertion was identified as a Class I transposable element (retrotransposon) due to the presence of flanking 429 bp long‐terminal repeats (LTRs) in direct orientation (Figure 4A; Table S5). The 2608 bp of internal sequence between the LTRs has no protein coding sequences nor long open reading frames, suggesting this element is non‐autonomous and relies on another transposable element for transposition. Its small (<4 kb) size defines it as a terminal repeat in miniature (TRIM) element (Mhiri et al., 2022) with previously observed four nucleotide LTR end conservation that is a feature of these TEs in other eudicot families (Figure 4A–B) (Yin et al., 2017). We subsequently named this non‐autonomous LTR retrotransposon that inserted into *AUX/1AA16*_*mut*_ *Outlaw*. To search for similar elements that may have functioned as the donor site from the same TE family, we aligned the insertion from the *de novo* assembly built from a dicamba‐resistant plant to the reference genome built from a herbicide‐sensitive plant. This identified a near perfect match (99.9% identity) 1 Mbp away from *AUX/IAA16* on chromosome 4 and another match (96.7% identity, with a 101 bp deletion that removes the TRIM terminal ‘TG’ motif) on chromosome 2 in both assemblies (Figure 4B; Table S6). Because the site on chromosome 4 is the most similar across the genome and retrotransposons use a ‘copy and paste’ mechanism of transposition, we hypothesize this to be the donor location for the retrotransposon inserted into *AUX/IAA16*_*Mut*_. A maximum likelihood phylogenetic tree supports this idea, as the sequences from both assemblies form a distinct clade from those on chromosome 2 (Figure S1). When comparing the insertion in *AUX/IAA16*_*Mut*_ with its donor site on chromosome 4, there is one single‐nucleotide polymorphism (SNP) and two single base pair insertions (Table S6). These insertions are found in homopolymer runs of at least 9 bp and are likely artifacts of the sequencing technology (Espinosa et al., 2024). In addition, the 429 bp LTR sequences of the insertion in *AUX/IAA16*_*Mut*_ are 100% identical. This similarity suggests very recent transposition. Given the approximated rate of mutation in *A. thaliana* of 7 × 10^−9^ base substitutions per site per generation (Ossowski et al., 2010), we estimate a new single base substitution to be generated in either one of these 429 bp regions approximately every 166 500 generations. Thus, we date this transposition event to have occurred within the last ~166 500 years. This estimation is very crude, but it suggests that the insertion into *AUX/IAA16*_*Mut*_ is extremely young in the context of evolutionary time. In support of the recent insertion into *AUX/IAA16*, the *Outlaw* insertion has perfectly identical 5 bp target site duplications (TGGCC; Figure 4A). We conclude that *Outlaw* is a very recent insertion of a TRIM element. Future research using collections of kochia in its native and invasive range and examining conservation in the linkage block surrounding *AUX/IAA16* could test whether this adaptation is the consequence of selection on standing genetic variation or genetic diversity that was generated in the time of modern agriculture (Kreiner et al., 2022).

### *AUX/* *IAA16*_*Mut*_ expression is sufficient for dicamba resistance in *A. thaliana*

**Figure 5:**
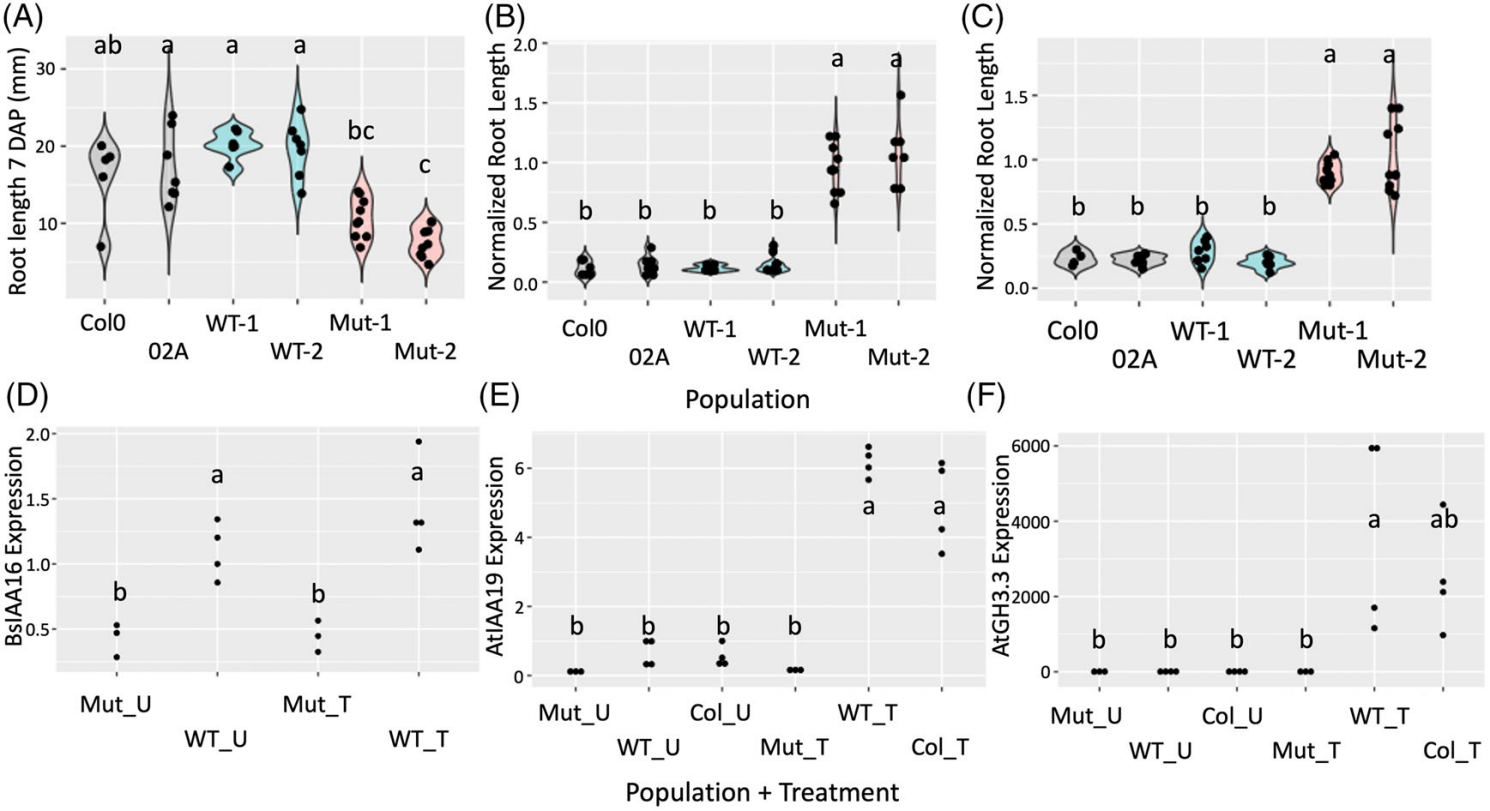
Evaluation of *Bassia scoparia AUX/IAA16* alleles in *Arabidopsis thaliana*. (A) Root length of plants with no transgene (Col0 and 02A; gray fill); plants expressing *AUX/IAA16*_*WT*_ (populations starting with WT; blue fill); and plants expressing *AUX/IAA16*_*Mut*_ (populations starting with Mut; red fill) when grown on phytoagar media. Proportional root length (compared with ethanol‐only control) of the same populations (coloring as in A) when grown on media containing 5 μM dicamba (B) or 0.5 μM IAA (C). Relative expression of the *AUX/IAA16* transgene (D) and the auxin response genes *AtAUX/IAA19* (E) and *AtGH3.3* (F) in plants expressing *AUX/IAA16*_*Mut*_ (Mut), *AUX/IAA16*_*WT*_ (WT), or no transgene (Col0) that were either treated (T) or untreated (U) with 140 g dicamba ha^−1^ 6 h after treatment. Each observation is represented by a black dot. Letters in all panels represent Tukey's HSD groups (alpha = 0.1).

To demonstrate the role of *AUX/IAA16*_*Mut*_ in dicamba resistance, stable transgenic lines of *A. thaliana* expressing *AUX/IAA16*_*WT*_ or *AUX/IAA16*_*Mut*_ under the CaMV35S promoter and OCS terminator were generated. When seeds were grown on phytoagar plates without herbicide, Arabidopsis expressing *AUX/IAA16*_*Mut*_ grew shorter roots compared with those expressing *AUX/IAA16*_*WT*_ or with no transgene (Figure 5A; Figure S2). This is evidence of a likely fitness cost associated with disrupting normal auxin signaling in roots due to mutant Aux/IAA expression as seen by de Figueiredo, Küpper, et al. (2022) and LeClere et al. (2018). When grown on media containing 5 μM dicamba, only seedlings expressing *AUX/IAA16*_*Mut*_ were able to grow and had significantly longer roots than seedlings expressing *AUX/IAA16*_*WT*_ or no transgene when normalized to the length of roots grown on solvent‐only media (Figure 5B; Figure S2). A similar result was observed when seedlings were grown on media containing 0.5 μM IAA (Figure 5C), suggesting this variant of AUX/IAA16 also reduces the perception of natural auxin. No seeds grew roots on media containing 5 or 0.5 μM 2,4‐D.

To confirm the results from root growth assays that indicate *AUX/IAA16*_*Mut*_ is sufficient for dicamba resistance, plants of select T_3_ lines either expressing *AUX/IAA16*_*WT*_, *AUX/IAA16*_*Mut*_, or no transgene were grown in the greenhouse and treated with a foliar spray of 140 g dicamba ha^−1^. Only one of the lines expressing *AUX/IAA16*_*Mut*_ showed less visual injury from dicamba than the other lines tested and appeared stunted and darker green compared with the other lines even when no dicamba treatment was administered (Figure S3). Quantitative polymerase chain reaction (PCR) indicated slightly higher expression of *AUX/IAA16* in the *AUX/IAA16*_*WT*_ line compared with the *AUX/IAA16*_*Mut*_ line (Figure 5D). However, we believe this difference in expression levels is not large enough to suggest RNA silencing in the *AUX/IAA16*_*WT*_ line. Plants expressing *AUX/IAA16*_*Mut*_ had no detected induction of auxin response genes *AtAUX/IAA19* or *AtGH3.3* 6 h after dicamba treatment, while the *AUX/IAA16*_*WT*_ and wild‐type lines showed marked increases in expression (Figure 5E,F). These genes are known to have increased expression following exposure to natural or synthetic auxins, and their expression serves as a proxy for auxin perception and signaling (de Figueiredo, Küpper, et al., 2022; Gleason et al., 2011; Pettinga et al., 2018; Tatematsu et al., 2004).

### Reduced binding of *AUX/* *IAA16*_*Mut*_ to auxin receptor protein TIR1 provides a molecular explanation of resistance

To provide a molecular explanation of the role of *AUX/IAA16*_*Mut*_ in auxin resistance, we measured binding of the Arabidopsis auxin receptor protein AtTIR1 to wild‐type and mutant AUX/IAA16 degron peptides using surface plasmon resonance (SPR; de Figueiredo, Küpper, et al., 2022; Prusinska et al., 2022). Binding was auxin‐dependent. In the presence of IAA, binding of the WT peptide was strong and persistent (i.e., peak intensity was high and dissociation of the co‐receptor complex was slow; Figure 3D). Binding of the mutant degron peptide was poorer in all respects. The binding amplitudes for the mutant peptide were lower than for the wild‐type (percent binding amplitudes of mutant versus wild‐type: IAA = 37%; 2,4‐D 18%; and dicamba 5%), and the complexes dissociated more rapidly. Intensity measured during the wash phase of the experiment was fitted to an exponential decay model to quantify and compare dissociation between peptides within treatments (Tables S7 and S8). Decay rates in binding were significantly faster for the mutant peptide compared with the wild‐type for all auxin analogs tested. In summary, the assembly of the co‐receptor complex with the mutant degron was greatly reduced, and when formed, its lifetime was shorter. This would lead to less AUX/IAA ubiquitination, higher concentrations of AUX/IAA16 remaining in the cell, and reduced auxin signal strength. Differences in expression of *AUX/IAA16* between populations may lead to differences in herbicide efficacy, and this is something not tested here. However, comparison of Arabidopsis lines with similar expression of wild‐type and mutant transgenes and these *in vitro* results reinforce our conclusion that sequence changes are chiefly responsible for resistance in this case.

### Fitness effects of the *AUX/* *IAA16*_*Mut*_ allele

**Figure 6:**
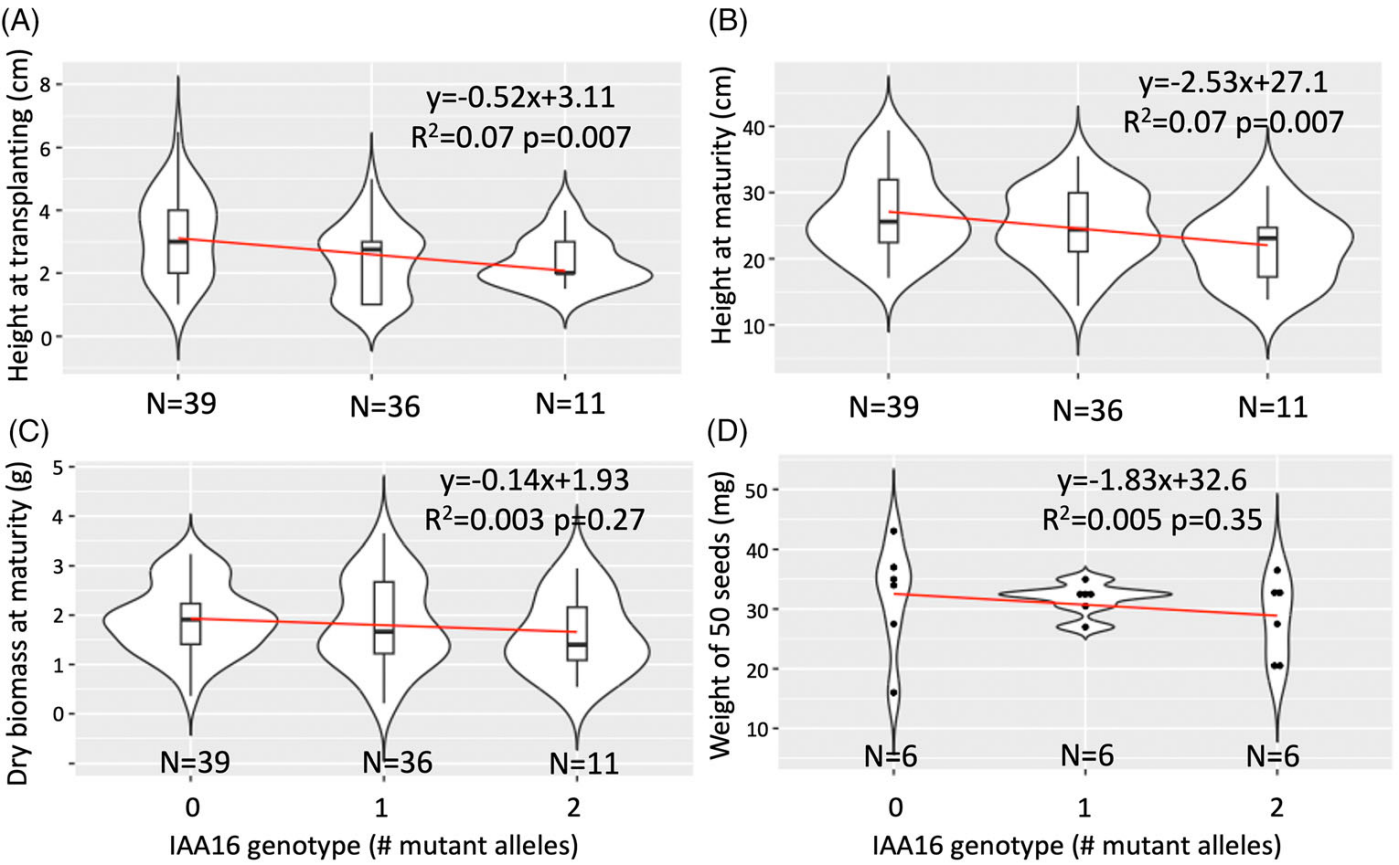
Pleotropic fitness effects of AUX/IAA16 genotypes in *Bassia scoparia*. Effect of *AUX/IAA16* genotype on plant height at transplanting (A) or maturity (B), dry biomass accumulation at maturity (C), or weight of 50 seeds (D) for *Bassia scoparia* plants from an F_3_ population segregating for dicamba resistance. Number of mutant alleles indicates homozygous wild‐type (0), heterozygous (1), or homozygous mutant (2). Linear models fitted to the data are plotted in red with equations, adjusted *R* ^2^, and *P*‐value of an *F*‐test testing the effect of genotype listed at the top right of each pane. Replicate number is listed at the base of each violin with either boxplots or data plotted as dots within each violin.

Because we observe lower binding of AUX/IAA16_Mut_ with TIR1 in the presence of natural auxin (Figure 3D), we expect this change to also confer a fitness penalty in the absence of synthetic auxin application (de Figueiredo, Küpper, et al., 2022; LeClere et al., 2018; Wu et al., 2021). To formally test this, kochia plants were grown from an F_3_ family that is segregating for the *AUX/IAA16*_*Mut*_ allele, and fitness traits were measured without the application of auxin. The ratio of genotypes significantly differed from the expected 1:2:1 (chi‐square test; *P* < 0.0001), with fewer than expected plants with the *AUX/IAA16*_*Mut*_ allele (Figure 6). All seedlings that emerged were used in the experiment, so we hypothesize that *AUX/IAA16*_*Mut*_ causes reduced germination. This may explain the over‐abundance of dead (herbicide‐sensitive) plants from our segregating F_3_ population described above used for QTL mapping. Further, *AUX/IAA16*_*Mut*_ was weakly but significantly associated with shorter plants, both at transplanting and at maturity (Figure 6A,B). However, *AUX/IAA16* genotype did not have a significant effect on above‐ground biomass (Figure 6C) or average seed weight (Figure 6D). Thus, while the *AUX/IAA16*_*Mut*_ allele is associated with reduced plant height, plants with this allele accumulate similar biomass and produce similarly weighted seeds. We acknowledge that sample size is not even across genotypes and that low sample size of the homozygous mutant allele places high leverage on these individuals. Nevertheless, these results suggest this herbicide resistance mechanism does slightly reduce plant fitness under normal (non‐selective) conditions, but not enough to purge it from the population.

Evidence of a fitness cost associated with herbicide resistance has implications for kochia management. For instance, if dicamba is not used for several years, the frequency of *AUX/IAA16*_*Mut*_ is expected to decrease. Future experiments to investigate allele frequency across generations under competition or abiotic stress will help clarify the real‐world ecological consequences of the *AUX/IAA16*_*Mut*_ allele (Karageorgi et al., 2025; Wu et al., 2018). Possible negative pleiotropic effects of *AUX/IAA16*_*Mut*_ may help limit the spread of this resistance allele and allow producers to better manage resistant populations that have already established. This will be especially important as high gene flow in kochia has facilitated the rapid spread of other herbicide resistance alleles (Ravet et al., 2021).

Taken together, our results further emphasize the importance of the glycine residue within the degron domain of AUX/IAA proteins in auxin signaling. They also reinforce the importance of *AUX/IAA16*, specifically, in the action of commonly used synthetic auxin herbicides. We describe the first instance of the insertion of a TRIM transposable element into the coding sequence of a gene to create an herbicide resistance allele. This work also highlights the contribution of transposable elements to genetic diversity in kochia, as the insertion of an unrelated mobile genetic element is also associated with gene duplication resulting in glyphosate resistance in this species (Hall et al., 2025; Patterson et al., 2019; Ravet et al., 2021). These results highlight the amazing adaptability of weedy genomes and the remarkable ways genomes evolve in the face of strong selection pressures (Johnson et al., 2025; Montgomery et al., 2024).

## EXPERIMENTAL PROCEDURES

### Plant growth and herbicide treatment conditions

Unless otherwise noted, all plants were grown in greenhouses at Colorado State University in 3.8‐cm by 3.8‐cm by 5.8‐cm pots containing fine‐grade potting mix (Fafard #2‐SV; American Clay Works, Denver, CO, USA). Temperatures were held between 22°C and 24°C, and supplemental light was provided by liquid halogen grow lights to ensure a photoperiod of 14/10 h. Herbicide applications were made using a moving overhead single‐nozzle sprayer (DeVries Manufacturing, Hollandale, MN, USA) calibrated to deliver 187 L ha^−1^. Engenia (BASF, Research Triangle Park, NC, USA) was used for dicamba applications, and Clean Amine (Loveland Products, Inc., Loveland, CO, USA) was used for 2,4‐D applications. For kochia, herbicide applications took place when plants were 10–15 cm tall, and for Arabidopsis, plants were sprayed when they had 10–12 leaves and before bolting.

### Plant material

The M32 population of kochia was collected near Akron, Colorado, as part of an herbicide resistance survey conducted between 2012 and 2014 (Westra et al., 2019). Seeds from this field collection were grown, and the resulting plants were treated with 560 g dicamba ha^−1^. Survivors were open‐pollinated, and their seed was combined to form a composite dicamba‐resistant seed lot. Approximately, 50 seeds from this combined lot were grown and treated with 560 g dicamba ha^−1^. The individual with the least herbicide injury was used as a male parent in a biparental cross with an individual from the population 7710, an inbred, herbicide‐susceptible population (Preston et al., 2009) that was used to generate the reference genome assembly for kochia (Hall et al., 2025; Patterson et al., 2019). To make the cross, immature flowers of the dicamba‐sensitive plant were emasculated using forceps under a microscope, and the emasculated flowers were labeled. The two plants were then grown to maturity together in a pollen‐exclusion tent. Seeds from the dicamba‐sensitive plant were collected and grown; the resulting plants were treated with 560 g dicamba ha^−1^. A Kompetitive Allele Specific PCR (KASP) assay that detects *AUX/IAA16*_*Mut*_ was used to confirm survivors as hybrids (primer sequence and thermocycler conditions found in Table S7). Several confirmed hybrid plants were grown in pollen‐exclusion tents and self‐pollinated to create several F_2_ families. Plants from two F_2_ families (each from a different F_1_ hybrid) were grown, but because of low seed production in the F_1_ generation, plants were allowed to self‐pollinate with no selection to produce F_3_ families. Each selected F_3_ family segregated for dicamba resistance and consisted of thousands of seeds.

### Dose–response

Plants were grown from the 7710 (Preston et al., 2009) and 9425 (LeClere et al., 2018) populations as well as from the M32 composite resistant seed lot. Plants were treated with one of several rates of dicamba. These rates included 0, 8.75, 17.5, 35, 70, 140, 280, 560, and 1120 g dicamba ha^−1^ for the herbicide‐sensitive population and 0, 70, 140, 280, 560, 1120, 2240, and 4480 g dicamba ha^−1^ for the herbicide‐resistant populations. Six uniform plants were used for each population at each herbicide rate before being returned to the greenhouse. Visual injury and survival were rated 21 DAT. The drc package v3.0‐1 (Ritz et al., 2015) in R v4.0.2 (R Core Team, 2020) was used to fit the injury data to a two‐parameter log‐logistic dose–response curve. Significant differences in the ED_50_ parameter were determined using the compParm function from the drc package (Ritz et al., 2015).

### Herbicide absorption and translocation profiles

Kochia plants from M32, 7710, and 9425 were germinated in a growth chamber (60% relative humidity, 21°C/18°C, and 16/8 h photoperiod) in potting soil and transplanted to fine sand when they reached ~3 true leaves. The plants were irrigated with fertilizer until the plants reached 10 cm in height. At this point, aluminum foil was used to cover the second youngest fully expanded leaf while the plants were sprayed with 560 g dicamba ha^−1^. After spraying, the aluminum foil was removed, the covered leaf was then marked and treated with 10 μl of a ^14^C‐labeled dicamba solution (total radioactivity of 3.33 KBq or 200 000 dpm per plant). Plants were returned to the growth chamber until sampling at 3, 6, 12, 24, 48, 96, and 192 h after treatment. The treated leaf, remaining above‐ground tissue, and below‐ground tissue of three biological replicates were separated for each population at each time point. The treated leaf was washed in 10 ml 10% methanol + 1% NIS, and radioactivity in this wash solution was quantified in 10 ml of scintillation mixture (Ecoscint XR, National Diagnostics, Atlanta, GA, USA) using liquid scintillation spectrometry (Packard Tricarb 2300TR, Packard Instrument Co., Meriden, CT, USA). Plant tissue was dried in an oven at 60°C for at least 14 days before oxidation in a biological oxidizer (OX500; RJ Harvey Instrument Co., Tappan, NY, USA) followed by radioactivity measurement by liquid scintillation spectrometry. Absorption time series data was fitted to a rectangular hyperbolic model, and the parameters ‘absorption max’ (*A*_max_) and ‘time to 90% absorption’ (*t*_90_) were estimated and compared in R (Kniss et al., 2011; R Core Team, 2020; Ritz et al., 2015). Because the time series data for translocation of dicamba outside of the treated leaf did not fit any of the models proposed by Kniss et al. (2011), *t*‐tests were used to determine if the amount of dicamba retained in the treated leaf was different between the M32 and 7710 populations at each time point.

### QTL mapping

Plants from two distinct F_3_ families derived from separate biparental crosses described above were grown and treated with 560 g dicamba ha^−1^. These two families that segregated for survival following dicamba treatment (named 4‐1‐1 and 4‐5‐10, each from a different F_1_ hybridization event) were used for QTL mapping. About 285 plants from the F_3_ family 4‐5‐10 were grown, and a single young leaf was sampled and frozen in liquid nitrogen before treatment with 560 g dicamba ha^−1^. Survival and visual injury on a scale from 0 to 100 was rated for each plant 21 DAT, and DNA was extracted from each plant (including the original parents of the cross) using the CTAB method (Doyle & Doyle, 1990). The concentration of each DNA sample was quantified using the Qubit™ dsDNA BR kit (Thermo Scientific, Waltham, MA, USA) and diluted to 20 ng μl^−1^. A double‐digest restriction site‐associated DNA sequencing (ddRADseq) protocol was developed in conjunction with the University of Minnesota Genomics Center (Minneapolis, MN, USA) by estimating the number of RAD sites across the reference genome (Patterson et al., 2019) for several enzyme combinations and then empirically testing the BtgI‐TaqI combination on several samples at multiple sequencing depths. DNA from the two original parents and 103 plants from the 4‐5‐10 F_3_ family were used for ddRADseq with BtgI and TaqI as the restriction enzymes with a target of 4 million 150 bp single‐end reads per sample. Library prep and sequencing were completed by the University of Minnesota Genomics Center.

Reads from each sample were passed through a variant calling pipeline that can be found at github.com/JMont12/dicamba_kochia. Briefly, raw reads were trimmed with Trimmomatic v0.36 (Bolger et al., 2014) and aligned to the reference genome of kochia (Hall et al., 2025) using the burrows‐wheeler aligner v0.7.17 (Li & Durbin, 2009). The alignments were processed with samtools v1.15 (Li et al., 2009) and passed to GATK v4.2.0 (Poplin et al., 2018; Van der Auwera & O'Connor, 2020) to call variants and filter them based on depth, quality, and strand bias. Variants were further filtered using custom scripts to only include biallelic SNPs that were homozygous and different between the two original parents. Filtered variants and phenotype data were used to conduct a genome scan within the qtl2 package in R v0.32 (Broman et al., 2019). Because a permutation test for significance was not possible, a significance threshold was established through a Bonferroni correction of alpha = 0.05 considering the number of tests to equal the number of filtered markers used in the scan, then converting the adjusted *P*‐value to LOD as described by Nyholt (2000). The find_peaks function from the qtl2 package in R was used to determine a 95% confidence interval of where the causal variant is contained. The genotype around the QTL discovered on chr4 was determined for 210 plants from another F_3_ family (4‐1‐1) using the KASP assay developed to detect the *AUX/IAA16*_*Mut*_ allele (primer sequence and thermocycler conditions in Table S9). A simple linear model was fitted using visual injury 21 DAT as the dependent variable and *AUX/IAA16* genotype as the independent variable and plotted using ggplot2 v3.4.4 (Wickham, 2016) in R (R Core Team, 2020).

Similarly, to quantify the effect of *AUX/IAA16* genotype on fitness traits, ~300 seeds of an F_3_ family segregating for the *AUX/IAA16* locus were sown into damp soil. Three weeks after planting, all emerged seedlings were transplanted into individual pots (10.5 × 10.5 × 12 cm) filled with potting soil and irrigated evenly throughout their lifecycle. Plants were spaced evenly, watered sufficiently, and not fertilized to reduce competition and environmental variation. Plant height was measured at transplanting and maturity. After allowing the plants to senesce naturally, above‐ground biomass was collected and weighed. For a subset of plants, seeds were harvested and a random sample of 50 seeds was used to determine average seed weight for each sample. During development, a small amount of leaf tissue was collected from each plant and used to determine the genotype of the *AUX/IAA16* locus using the KASP assay described above. Genotype and phenotype information was used to fit linear models and plotted using ggplot in R.

### *AUX/IAA16* sequencing and retrotransposon characterization

Total RNA was extracted using the Direct‐zol RNA Microprep kit (Zymo Research, Irvine, CA, USA) from individuals of the 4‐5‐10 selected F_3_ line that were homozygous for either parental allele of *AUX/IAA16* based on the results of KASP testing. This RNA was used to generate cDNA libraries for each sample using the ProtoScript® II First Strand cDNA Synthesis Kit (New England Biolabs, Ipswich, MA, USA). Primers that bind to the beginning and end of the *AUX/IAA16* gene (Table S10) were used with the EconoTaq PLUS Green kit (LGC, Teddington, Middlesex, UK) to amplify the full‐length coding sequence. The product of each PCR reaction was run on a 1% agarose gel to ensure a single product, then sent to Azenta (Burlington, MA, USA) for Sanger sequencing using the forward and reverse primers.

To determine the genomic sequence around the *AUX/IAA16*_*Mut*_, 2 g of dark‐treated leaf tissue from a plant that was homozygous for *AUX/IAA16*_*Mut*_ was sampled and flash frozen in liquid nitrogen. This tissue was sent to Corteva Agriscience Center for Genome Excellence (Johnston, IA, USA) for whole genome PacBio HiFi sequencing to a depth of ~30×. The resulting reads were assembled with HiFiasm v0.19.5 (Cheng et al., 2021), and *AUX/IAA16* was located by BLAST v2.6.0 (Camacho et al., 2009). To find similar elements to the one inserted in *AUX/IAA16*_*Mut*_, the sequence of the 429 bp LTRs of the insertion was aligned to the kochia reference genome assembly (Hall et al., 2025) and to the newly created assembly described above using BLAST (Camacho et al., 2009). Alignment of the TRIM TE element in *AUX/IAA16*_*Mut*_ and two similar elements was done through MAFFT v7 (Katoh & Standley, 2013) and visualized through Benchling Biology Software 2025.

### Development of transgenic *A. thaliana* lines

The coding sequence of *AUX/IAA16*_*Mut*_ and *AUX/IAA16*_*WT*_ was amplified from cDNA libraries discussed above using the PrimeSTAR MAX DNA Polymerase kit (Takara Bio USA, Inc., San Jose, CA, USA) and primers with tails that complement restriction sites in the expression vector *pFGC5941* (primer sequences and thermocycler conditions in Table S11). Empty *pFGC5941* was digested with *Asc*I and *Bam*HI (New England Biolabs, Ipswich, MA, USA), and the *AUX/IAA16* amplicons were ligated into the digested vector using the In‐Fusion Cloning kit (Takara Bio USA, Inc., San Jose, CA, USA). This vector expresses its gene of interest using the CaMV 35S promoter and OCS terminator. Plasmid sequencing ensured correct assembly of expression vectors (Plasmidsaurus, Eugene, OR, USA). Vectors containing *AUX/IAA16*_*Mut*_ or *AUX/IAA16*_*WT*_ were transformed into the GV3101 strain of *Agrobacterium tumefaciens* and subsequently transfected into the Col‐0 genotype of *A. thaliana* using the floral dip method (Clough & Bent, 1998). Transformants were selected by spraying T_1_ seedlings with BASTA herbicide (BASF, Research Triangle Park, NC, USA) and confirmed with PCR for the transgene (primers and thermocycler conditions found in Table S12). BASTA resistance was used as a selectable marker because the expression vector used here (*pFGC5941*) contains the *bar* gene, which endows BASTA resistance. Confirmed transformants were inbred for two generations, and T_3_ populations fixed for the transgene were identified by spraying seedlings with BASTA herbicide and PCR for the transgene.

### Effect of dicamba on transgenic Arabidopsis lines

Seeds of Col‐0 and of fixed T_3_ lines of Arabidopsis, each from a different transformation event, were gas sterilized and plated on media containing synthetic or natural auxin as described by de Figueiredo, Küpper, et al. (2022). Plates were incubated in a growth chamber with a yellow light filter to prevent IAA degradation. Ten seeds were used per population for each treatment. Root growth was measured 7 days after moving plates to growing conditions. Tukey's HSD was used to detect significant differences in root length between all tested lines for each treatment. Results were plotted with ggplot2 in R.

Plants from select T_3_ lines were grown to the 10‐leaf stage and either untreated or treated with 140 g dicamba ha^−1^. Four plants of each population were used for each treatment. Photos were taken 21 DAT to illustrate visual injury. Young leaf tissue was collected from treated and untreated plants 6 h after treatment. Total RNA was extracted from these leaf samples using the Direct‐zol RNA Microprep kit (Zymo Research, Irvine, CA, USA), and cDNA libraries were generated using the ProtoScript® II First Strand cDNA Synthesis Kit (New England Biolabs, Ipswich, MA, USA). Following the methods of de Figueiredo, Küpper, et al. (2022), relative expression was quantified for the *AUX/IAA16* transgene (primers and thermocycler conditions available in Table S13) and the auxin response genes *AtAUX/IAA19* and *AtGH3.3* using *AtCyclophilin* as a reference gene and the 2^−ΔΔCt^ method.

### *In silico* mutagenesis of *AUX/IAA* variants

The crystal structure of the degron of AUX/IAA7 bound to TIR1 in the presence of 2,4‐D (Entry ID; 2p1n) was downloaded from the RCSB Protein Data Bank (Tan et al., 2007). Mutagenesis of the G127 residue was conducted and visualized in PyMOL.

### Receptor protein preparation and surface plasmon resonance measurement of auxin binding

Receptor protein (AtTIR1) was purified and the SPR assays performed following the methods described in Prusinska et al. (2023). The SPR chip (Series S SA) was loaded with our reference negative control degron peptide mAUX/IAA7 (mutated Arabidopsis AUX/IAA7); channel 2 with positive control degron peptide AUX/IAA7; channel 3 with AUX/IAA16_WT_ degron peptide (biotin‐AKTQVVGWPPVRAFRKN‐amide) and channel 4 with AUX/IAA16_Mut_ degron peptide (biotin‐AKSIKFPTWPPVRAFRKN‐amide). Similar peptide loads were recorded for all four channels. Purified receptor was passed over all four channels simultaneously in parallel in PBS buffer +0.01% Tween20, with or without test auxin (IAA; 2,4‐D; or dicamba) at a concentration of 50 μM. Binding was recorded for 120 sec, and dissociation for 180 sec. Data were visualized in BiaEvaluation software. Binding intensity for the wash phase of the experiment (after 165 sec) was extracted and fitted to exponential decay models (EXD.3) for each peptide/auxin combination using the drc package in R (Ritz et al., 2015). A *t*‐test was used within the compParm function to compare the parameter estimates for the rate of decay between peptides for each auxin analog (Tables S7 and S8).

## CONFLICT OF INTEREST

The authors have not declared a conflict of interest.

## Supporting information


**Figure S1.** Maximum Likelihood tree showing relatedness of retrotransposons similar to the one inserted in *IAA16*. Names correspond to the descriptions in the caption of Table S5.
**Figure S2.** Representative photos of root growth assays of wildtype (Col0 and 02A) and transgenic Arabidopsis either expressing the wildtype (subscript WT) or mutant (subscript M32) allele of BsAUX/IAA16. Seeds plated on phytoagar with no herbicide (left) or 5 uM dicamba (right).
**Figure S3.** *Arabidopsis thaliana* plants (genotype Col 0) plants expressing *BsIAA16* alleles (*BsIAA16*_*WT*_, 7710 2‐6‐2; *BsIAA16*_*MUT*_, M32 2‐3‐5) or with no transgene (02A) either untreated or treated with 140 g dicamba ha^−1^. Photo taken 14 days after treatment.
**Table S1.** Parameter estimates for two‐parameter log‐logistic dose response curves for M32, 9425, and 7710 kochia populations in response to dicamba. The equation fitted for each population was $y=\frac{100}{1+b\left(\log\left(x\right)-\log\left({\mathrm{ED}}_{50}\right)\right)}$ where *y* is predicted visual injury (0–100) 21 days after dicamba treatment, *x* is the rate of dicamba in g ha^−1^, ED_50_ is the rate of dicamba required to cause 50% visual injury, and *b* is the slope of the curve at *x* = ED_50_.
**Table S2.** Parameter estimates for two‐parameter log‐logistic dose response curves for M32 and 7710 kochia populations in response to 2,4‐D. The equation fitted for each population was $y=\frac{100}{1+b\left(\log\left(x\right)-\log\left({\mathrm{ED}}_{50}\right)\right)}$ where *y* is predicted visual injury (0–100) 21 days after 2,4‐D treatment, *x* is the rate of 2,4‐D in g ha^−1^, ED_50_ is the rate of 2,4‐D required to cause 50% visual injury, and *b* is the slope of the curve at *x* = ED_50_.
**Table S3.** Parameter estimates for rectangular hyperbolic models fitted to herbicide absorption data for M32, 9425, and 7710 kochia populations. The equation fitted for each population was *y* = (*A*_max_ × *t*)/(0.11 × *t*_90_ + *t*) where *y* is percent of applied herbicide that was absorbed, *t* is time after herbicide application (in hours), *t*_90_ is the time required for 90% herbicide absorption, and *A*_max_ is the maximum amount of herbicide absorbed.
**Table S4.** CLUSTAL multiple protein sequence alignment of 7710 and M32 alleles of AUX/IAA16. The degron domain is bolded.
**Table S5.** Sequences of non‐autonomous Outlaw element. IAA16_insertion is the non‐autonomous element that is inserted in the IAA16 gene on chromosome 4. 100 bases on the 5′ and 3′ ends that are highlighted in yellow are not part of the Outlaw element but help to show genomic context.
**Table S6.** CLUSTAL alignment of transposable elements similar to the one in *AUX/IAA16* from the M32 population. Outgroup represents the version on chromosome 2. Donor represents the conserved version found on chromosome 4. IAA_insertion is the M32‐specific version inside of *AUX/IAA16*.
**Table S7.** Parameter estimates and their standard errors for three‐parameter exponential decay models predicting the binding intensity of mutant (Mut) and wildtype (WT) degron peptides measured during the wash phase of a surface plasmon resonance experiment. Binding intensity measured between TIR1 and degron peptides in the presence of one of several auxin analogs or DMSO as a negative control. The equation fitted for each peptide/auxin combination was $y_{t}=c+\left(d-c\right)e^{-t/\propto }$ where *y*_*t*_ is the predicted binding intensity at time *t*, *c* is the lower limit of binding intensity as *t* approaches infinity, *d* is the upper limit of binding intensity at the beginning of the experiment, and ∝ is the rate of decay. Smaller values of ∝ indicate faster decay. DMSO did not fit to the model due to lack of binding as expected for the negative control, so no equation parameters are presented for DMSO.
**Table S8.** Results of *t*‐tests for comparing the rate of decay in binding intensity for different degron/auxin combinations. Estimates greater than 1 indicate wildtype decay in binding is slower than mutant.
**Table S9.** KASP primer set for identification of M32 allele of IAA16. The primer that binds to the wildtype has a FAM tail (green) and the primer that binds the M32 allele has a HEX tail (orange). Thermocycler conditions are 94 C for 15 min; 10 cycles of 94 C for 20 sec, 61 C for 60 sec and decreasing 0.6 C each cycle; 40 cycles of 94 C for 20 sec, 55 C for 60 sec, quantifying FAM and HEX fluorescence at each round at 30 C.
**Table S10.** Polymerase chain reaction primers used to amplify *BsIAA16* from cDNA libraries. Thermocycler conditions were 95 C for 5 min; 35 cycles of 95 C for 15 sec, 60 C for 15 sec, 72 C for 1 min; 72 C for 5 min.
**Table S11.** Polymerase chain reaction primers used to clone *BsAUX/IAA16* into pFGC5941. Thermocycler conditions were 95 C for 5 min; 35 cycles of 95 C for 15 sec, 60 C for 15 sec, 72 C for 1 min; 72 C for 5 min. Tails used for In‐Fusion cloning are colored in green.
**Table S12.** Polymerase chain reaction primers used to amplify a section of pFGC5941 that contains the transgene of interest. Thermocycler conditions were 95 C for 5 min; 35 cycles of 95 C for 15 sec, 60 C for 15 sec, 72 C for 2 min; 72 C for 5 min. Positive samples should show an amplicon of ~400 bp + the length of your transgene.
**Table S13.** Quantitative PCR primers to quantify expression of various *Arabidopsis thaliana* and *Bassia scoparia* genes. Thermocycler conditions for all primer sets are 95 C for 3 min; 40 cycles of 95 C for 15 sec, 60 C for 30 sec, quantifying SYBR fluorescence after each round at 60 C.
**Table S14.** Phenotypic response of kochia from a F_3_ populations segregating for herbicide resistance. Visual injury was rated 21 days after treatment on a scale from 0 to 100 and survival was noted (A = alive, D = dead).

## Data Availability

The RAD sequencing data from the segregating F3 line used in the mapping project is available through NCBI, BioSample IDs SAMN42890277‐SAMN42890594 and SRA accession numbers SRR30019316‐SRR30019633. Phenotypic data can be found in Table S12. PacBio HiFi sequencing used to generate the *de novo* genome assembly is available through NCBI under BioSample ID SAMN43525227 and SRA accession number SRR30575711. All sequence data is associated with NCBI BioProject PRJNA1141446.
