# Supplementary material for: A transposable element insertion in AUX/IAA16 disrupts splicing and causes auxin resistance in Bassia scoparia
Source: Plant J. 2025 Jul 20;123(2):e70339. doi: 10.1111/tpj.70339 (PMC12277486; doi:10.1111/tpj.70339)
Supplement: Supplementary file 1 — Figure S1. Maximum Likelihood tree showing relatedness of retrotransposons similar to the one inserted in IAA16. Names correspond to the descriptions in the caption of Table S5. Figure S2. Representative photos of root growth assays of wildtype (Col0 and 02A) and transgenic Arabidopsis either expressing the wildtype (subscript WT) or mutant (subscript M32) allele of BsAUX/IAA16. Seeds plated on phytoagar with no herbicide (left) or 5 uM dicamba (right). Figure S3. Arabidopsis thaliana plants (genotype Col 0) plants expressing BsIAA16 alleles (BsIAA16 WT , 7710 2‐6‐2; BsIAA16 MUT , M32 2‐3‐5) or with no transgene (02A) either untreated or treated with 140 g dicamba ha−1. Photo taken 14 days after treatment. Table S1. Parameter estimates for two‐parameter log‐logistic dose response curves for M32, 9425, and 7710 kochia populations in response to dicamba. The equation fitted for each population was y=1001+blogx−logED50 where y is predicted visual injury (0–100) 21 days after dicamba treatment, x is the rate of dicamba in g ha−1, ED50 is the rate of dicamba required to cause 50% visual injury, and b is the slope of the curve at x = ED50. Table S2. Parameter estimates for two‐parameter log‐logistic dose response curves for M32 and 7710 kochia populations in response to 2,4‐D. The equation fitted for each population was y=1001+blogx−logED50 where y is predicted visual injury (0–100) 21 days after 2,4‐D treatment, x is the rate of 2,4‐D in g ha−1, ED50 is the rate of 2,4‐D required to cause 50% visual injury, and b is the slope of the curve at x = ED50. Table S3. Parameter estimates for rectangular hyperbolic models fitted to herbicide absorption data for M32, 9425, and 7710 kochia populations. The equation fitted for each population was y = (A max × t)/(0.11 × t 90 + t) where y is percent of applied herbicide that was absorbed, t is time after herbicide application (in hours), t 90 is the time required for 90% herbicide absorption, and A max is the maximum amount of herbi [file TPJ-123-0-s001.docx]

Supplemental Information

Figure S1. Maximum Likelihood tree showing relatedness of retrotransposons similar to the one inserted in *IAA16*. Names correspond to the descriptions in the caption of Table S5.


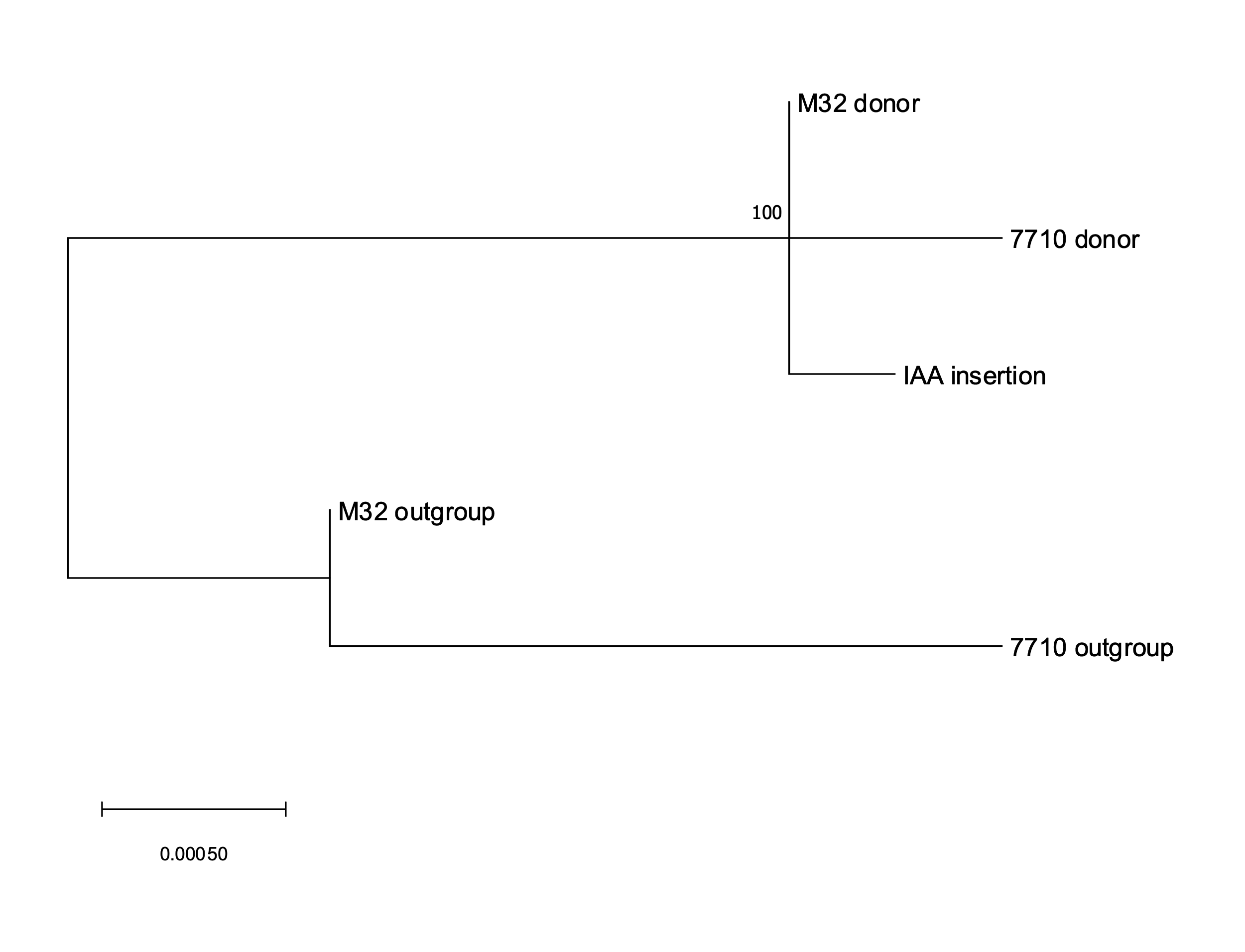


Figure S2. Representative photos of root growth assays of wildtype (Col0 and 02A) and transgenic arabidopsis either expressing the wildtype (subscript WT) or mutant (subscript M32) allele of BsAUX/IAA16. Seeds plated on phytoagar with no herbicide (left) or 5 uM dicamba (right).


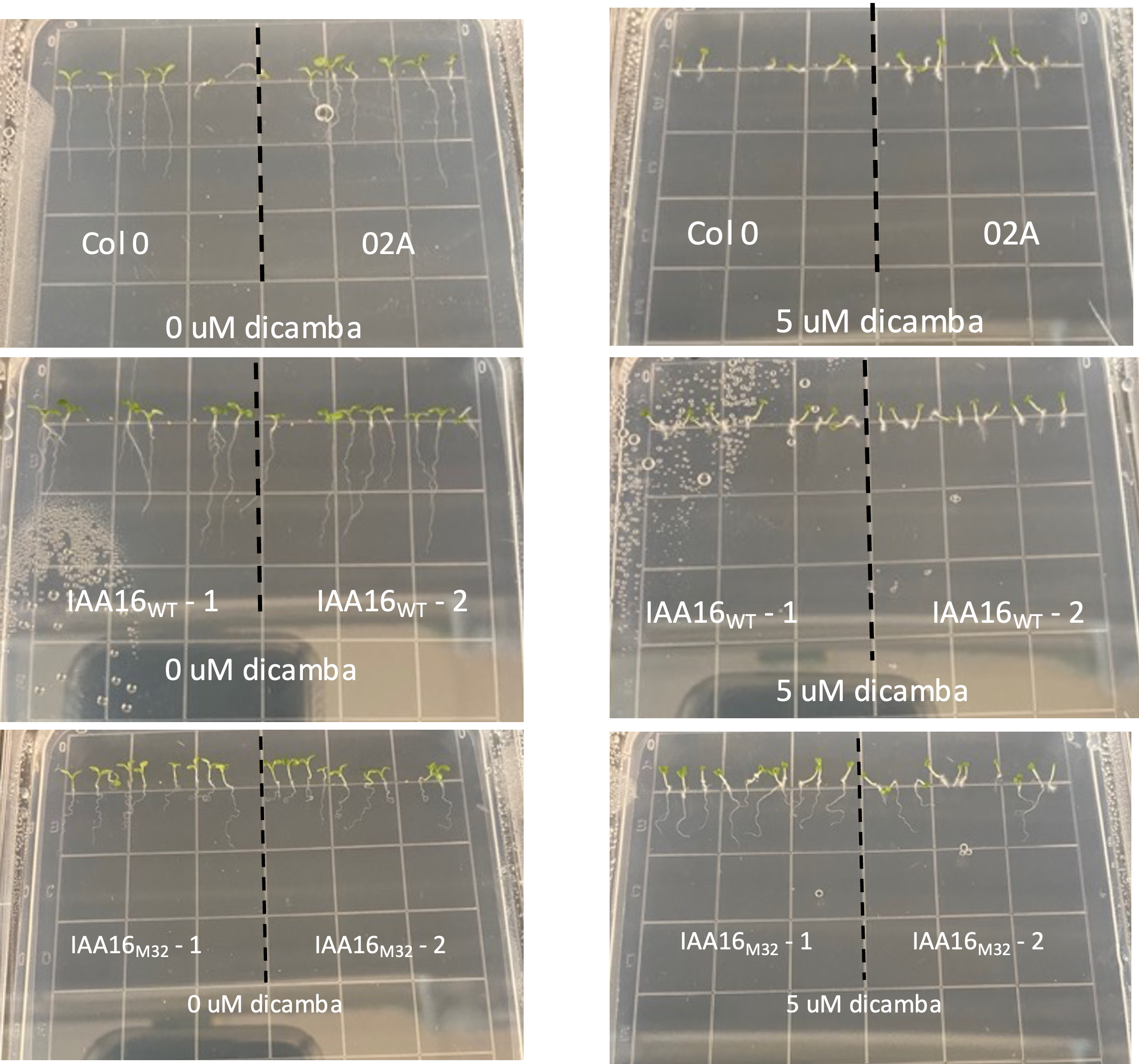


Figure S3. *Arabidopsis thaliana* plants (genotype Col 0) plants expressing *BsIAA16* alleles (*BsIAA16_WT_*, 7710 2-6-2; *BsIAA16_MUT_*, M32 2-3-5) or with no transgene (02A) either untreated or treated with 140 g dicamba ha^-1^. Photo taken 14 days after treatment.

Table S1. Parameter estimates for 2-parameter log-logistic dose response curves for M32, 9425, and 7710 kochia populations in response to dicamba. The equation fitted for each population was $y=\frac{100}{1+b(\log\left( x \right)-\log\left( ED50 \right))}$ where *y* is predicted visual injury (0-100) 21 days after dicamba treatment, *x* is the rate of dicamba in g ha^-1^, *ED50* is the rate of dicamba required to cause 50% visual injury, and *b* is the slope of the curve at x=ED50.

| Parameter | Estimate | Std. Error |
| --- | --- | --- |
| Slope:7710 | -2.9 | 0.3 |
| Slope:M32 | -4.5 | 0.9 |
| Slope:9425 | -2.1 | 0.3 |
| ED50:7710 | 130.6 | 16.9 |
| ED50:M32 | 988.3 | 27.2 |
| ED50:9425 | 1370.4 | 20.9 |

Table S2. Parameter estimates for 2-parameter log-logistic dose response curves for M32 and 7710 kochia populations in response to 2,4-D. The equation fitted for each population was $y=\frac{100}{1+b(\log\left( x \right)-\log\left( ED50 \right))}$ where *y* is predicted visual injury (0-100) 21 days after 2,4-D treatment, *x* is the rate of 2,4-D in g ha^-1^, *ED50* is the rate of 2,4-D required to cause 50% visual injury, and *b* is the slope of the curve at x=ED50.

| Parameter | Estimate | Std. Error |
| --- | --- | --- |
| Slope:7710 | -1.5 | 0.1 |
| Slope:M32 | -2.3 | 0.4 |
| ED50:7710 | 543.1 | 21.2 |
| ED50:M32 | 2720.7 | 307.8 |

Table S3. Parameter estimates for rectangular hyperbolic models fitted to herbicide absorption data for M32, 9425, and 7710 kochia populations. The equation fitted for each population was 𝑦 = (𝐴𝑚𝑎𝑥 × 𝑡)/(0.11 × 𝑡90 + 𝑡) where *y* is percent of applied herbicide that was absorbed, *t* is time after herbicide application (in hours), *t90* is the time required for 90% herbicide absorption, and *Amax* is the maximum amount of herbicide absorbed.

| Parameter | Estimate | Std. Error |
| --- | --- | --- |
| Amax:7710 | 66.8 | 5.6 |
| Amax:M32 | 64.4 | 3.9 |
| Amax:9425 | 45.7 | 5.3 |
| t90:7710 | 27.2 | 9.5 |
| t90:M32 | 8.3 | 4.1 |
| t90:9425 | 19.3 | 14.6 |

Table S4.

CLUSTAL multiple protein sequence alignment of 7710 and M32 alleles of AUX/IAA16. The degron domain is bolded.

7710 MLSNERDKYTIDFEETELRLGLGLGIGLAGAADGDQLAKNNNGKRGFSETEGDSSVDLKL

M32 MLSNERDKYTIDFEETELRLGLGLGIGLAGAADGDQLAKNNNGKRGFSETEGDSSVDLKL ************************************************************

7710 NLSSSTTTTASTTTTNTTATKTTAENVKESKLDKSVNSGVDQKLKEKVASTTADPAKPTP

M32 NLSSSTTTTASTTTTNTTATKTTAENVKESKLDKSVNSGVDQKLKEKVASTTADPAKPTP ************************************************************

7710 AKT-QVV**GWPPV**RAFRKNIVAAHKKTSDDQTDQKASSNAITSAAFVKVSMDGAPYLRKVD

M32 AKSIKFP**TWPPV**RAFRKNIVAAQKKTSDDQTDQKASSNAITSAAFVKVSMDGAPYLRKVD

**: :. ******************:*************************************

7710 LKLYKSYQDLSDALGKMFSSFTIGNCGSQGMKDFMNESKLIDLLNGSEYVPTYEDKDGDW

M32 LKLYKSYQDLSDALGKMFSSFTIGNCGSQGMKDFMNESKLIDLLNGSEYVPTYEDKDGDW ************************************************************

7710 MLVGDVPWEMFVGSCKRLRIMKGSEAIGLAPRAVEKCKNRS*

M32 MLVGDVPWEMFVGSCKRLRIMKGSEAIGLAPRAVEKCKNRS*

*****************************************

Table S5. Sequences of non-autonomous Outlaw element. IAA16_insertion is the non-autonomous element that is inserted in the IAA16 gene on chromosome 4. 100 bases on the 5’ and 3’ ends that are highlighted in yellow are not part of the Outlaw element but help to show genomic context.

>IAA16_insertion

CTCAAAGAAAGAAATGAAGATGTTTAGTCTAATTAAGTAAGTTAATCACGTAACTAATTCTTAATTGTTTGTTCTTCAGGACACAAGTTGTAGGTTGGCCTGTTGGGATTAAACCCTAATTCATATCAAATTAGTCGGTAATAATAGATCTGCAGAAGCATACCTGAATCCATGACGAAGATCGGCGGTGGGTTTTGATCTTCCAATTCTTTATGGCTCCTTAGGGTTTCTACTGATGATGGGATGTCAGTGAGAATAGGAAAACCATATGAATCGGGGACCATAACCCTTAATGTCTATTTATATACATAGACTCCTTCCTAATCCGCCCATCGTGAATAAGGAAAGGCCCATCGGTATCTACACAAATAAAAAACTGATCCCACACTCTATAAAAGACGTAAATAGGCCCAATAATAATTACTTAATTGGATCACTTTAGTTTTGGGCCACACCGTATGATAGCACATAATACAATTATAACTGAATTGCACACGTATTTGTATTTAGGTCCATAAAATTTCCAACAGTCTCCCACTTGGACCAAATACAATTACAACGTGTGTGATACTTGATAATTGCATTATACTTTATAACCTTATGAGCTCAAAATTGCTATCAAAATCTCAACGTCTTTAAACAATTCAGTCCATTAATTACATCAACACAGGATCAAAGAGATCTATGCTACATTTGCCGTAACCAGACCCATCAATGGTCACAATGTCAACATAATTAACGACATGAATCAAGCATGGGTGTGTAGCATGGAAATTACATACAAATGTGATCCAAGTATGCCTATTTCCAACTGGTCCACTGTAAACTTTGGTAAGATCAACAATATGATCTAATCGAAGAGTAAACCGAACCGAATACCTTATTTCTGCAGAAAATACCTTAAACCTTAATATCCGAATTAGAACTTCATCATACAAGCATCAACATAACAAACTCCCACTGAAACTGTATATCCTTAATACCTTAACTGGCATGACACACATGAATAATGTGCTCATGAAATACTTTAAGTATTTAACTTTAGTAAGCGGATCCGCAATAATGGAGTTGTATTATGATGCTTTAAGTACAATAATTTTACTCTTCAAACTTTCACAACACTAAAGCCATGATAAAAATTTAACCGTATTTGGATTTCAAGCTACCTTGACATCCACCAAAAACGGAGTCTAATAGCTGTAAGTTCTCTCTAATGGATCTCTTTAATGATACTTATTCTTGAGGTTGTTGAGTTTGCTCATTGGAATTTTCCTTTTGAGATCCTAAATAGGGGAATAAATCCTTATCTTCCCCCAAACTCAACATCCTCAAAGGACAAGCATCTATTGTATCCAAAAATTGATCCAATAGTGAGACTATAAAAAAAAATGTATCCTTTATTATTATCTTCATCAATATCTTTAGTGAAAGATGTCAAGTGAGCACTTTATATCTTTCATTGTTTCAATCTCTCTTCCTCCGAATCAACATGAGTAGTGAACTCATTTAGAGGTTCACTTCTCCATAACAACAATTTAGGAAGAGATTTAAAAACAAATGCACAAGCAAGTCGTTAGACTCCTCCAATTTAAGTGCCCTTAAATCATGAAACAATATCACAAATTTCCATAATGTGCTCCCTTAATTTATTTATTTTCCCCTTAATACCTTTTTCCCTTATGGAATTTAAGTCCAACAAGGTTGTACTCAATTCCCCTGATCTTTATAGCAAAACACTTTAAGCTCTTTAGAGAACAATTTGACATCTTTCTCTTTCATTTGGACATACATAATGTCCCCTAATAACTCCGAAATTGTACTTTAAATTGCACTTTACATGATCATATACGACCTATCCAATTTGAGCATTCTCACTTCTCCATATTCCCCTTTAGTAGGAGCACTAAAATCCATAAGAGTTGCGAGTGCTCAACTCGAAAAGCAAGGTCTGATCTAGATCCATGCAAAAAAAAAAAAAAAACCAAAACCTTCAATTTATTATCCTTAATCAGATTAATATATTGACAATGTATCCCATCATGTACAAGAGACCTAACACAACATTAATTTTTTAGTCTTTGGACAGAAAAATTAACTTGTAAGTGGTCCTCTTGGTGTAGTAATCAAATACTAACAATAAATTCATGTCAATGTTAAGCCTTTCTTTGGACCGACTTAACAACGCATGAAAAACCGAACAATTGTTACATATTTATTACCACAAGCATGTTTAATTTCCGGCCAAATAATAGCCTTCCTTTGGGCCGACCATTATCCGCATGAAAACTAAACACACACAGGTATCTCAATATTTCAAATTAATTGATCTACACAAAAGAGGTTACTTTGGCAACATATTGTTTCAATCAATTAATCCAAAATACTAGACCCAACTTAATAACCAAATTGCCAATTATTACAATCCGAAATATATGATGAATCCATAATTAGTCATGTGTTTATGAATCCATGACCTCAAATAGATAAGATGAAGCGCAAAATAAGTTGTAAACTGTAACACTAAAAATTAATCGATGGATTCATAATTCATAGCAACACAGCAGCGACTAACAATTGAATTAAGCAATAAATTGTTCTGTCTTATATATGGAGTAAGATTAAAATACCAAAGACTTTAACCACCAACACCCAGGCCAGGTCAAAAAATCAACCGAAATAAATAAAGTCAAGCTACATATATCTAGTGACTTTATCGATTCTAATCTTAAGAAAGGAATCACCAAATCAATGCCAAAACTAAAAAATAATCAGAACGATGACCAACCATATTTGAAGCACCCACAAAGCCTCATATGTCGAAATAAAATATCTGAAGTAAACTGTAAGGGATATTCAATTAAAGCATAATAGGGAATCAAAACCCAAATTAAATTTGGATGCTAACAAAGCCAAAAACATACACACACAACTGTCAACTGGATCGAATTGTGCATGTCAAATTGTCAATCATGACAATTGAGAAATAATTGATAACATACATCTGCCTTTTAGGCCTCGTCAATTAACAAATACTCAAACTCAATAGTTGATCACAAAACAAATTCGAAGTTCTGAAAGAAAAATACTTCGTACCAAATAATTTAATATGAAGAAGGAGATCCATAAAGAATTGGACTTGTTGGGATTAAACCCTAATTCATATCAAATTAGTCGGTAATAATAGATCTGCAGAAGCATACCTGAATCCATGACGAAGATCGGCGGTGGGTTTTGATCTTCCAATTCTTTATGGCTCCTTAGGGTTTCTACTGATGATGGGATGTCAGTGAGAATAGGAAAACCATATGAATCGGGGACCATAACCCTTAATGTCTATTTATATACATAGACTCCTTCCTAATCCGCCCATCGTGAATAAGGAAAGGCCCATCGGTATCTACACAAATAAAAAACTGATCCCACACTCTATAAAAGACGTAAATAGGCCCAATAATAATTACTTAATTGGATCACTTTAGTTTTGGGCCACACCGTATGATAGCACATAATACAATTATAACTGAATTGCACACGTATTTGTATTTAGGTCCATAAAATTTCCAACATGGCCACCCGTTAGAGCGTTCAGGAAGAACATCGTCGCGGCCCAAAAGAAGACGTCCGATGATCAAACTGATCAGAAGGCTTCTAGCAATGCCATCACAAGCGCC

Table S6. CLUSTAL alignment of transposable elements similar to the one in *AUX/IAA16* from the M32 population. Outgroup represents the version on chromosome 2. Donor represents the conserved version found on chromosome 4. IAA_insertion is the M32-specific version inside of *AUX/IAA16*.

IAA_insertion TGTTGGGATTAAACCCTAATTCATATCAAATTAGTCGGTAATAATAGATCTGCAGAAGCA

M32_donor TGTTGGGATTAAACCCTAATTCATATCAAATTAGTCGGTAATAATAGATCTGCAGAAGCG

7710_donor TGTTGGGATTAAACCCTAATTCATATCAAATTAGTCGGTAATAATAGATCTGCAGAAGCG

M32_outgroup T-----------------------------------------------------------

7710_outgroup T-----------------------------------------------------------

*

IAA_insertion TACCTGAATCCATGACGAAGATCGGCGGTGGGTTTTGATCTTCCAATTCTTTATGGCTCC

M32_donor TACCTGAATCCATGACGAAGATCGGCGGTGGGTTTTGATCTTCCAATTCTTTATGGCTCC

7710_donor TACCTGAATCCATGACGAAGATCGGCGGTGGGTTTTGATCTTCCAATTCTTTATGGCTCC

M32_outgroup ------------------------------------------CCAATTCTTTATGGCTCC

7710_outgroup ------------------------------------------CCAATTCTTTATGGCTCC

******************

IAA_insertion TTAGGGTTTCTACTGATGATGGGATGTCAGTGAGAATAGGAAAACCATATGAATCGGGGA

M32_donor TTAGGGTTTCTACTGATGATGGGATGTCAGTGAGAATAGGAAAACCATATGAATCGGGGA

7710_donor TTAGGGTTTCTACTGATGATGGGATGTCAGTGAGAATAGGAAAACCATATGAATCGGGGA

M32_outgroup TTAGGGTTTCTACTGATGATGGGATGTCAGTGAGAATAGGAAAACCATATAAATCGGGGA

7710_outgroup TTAGGGTTTCTACTGATGATGGGATGTCAGTGAGAATAGGAAAACCATATAAATCGGGGA

************************************************** *********

IAA_insertion CCATAACCCTTAATGTCTATTTATATACATAGACTCCTTCCTAATCCGCCCATCGTGAAT

M32_donor CCATAACCCTTAATGTCTATTTATATACATAGACTCCTTCCTAATCCGCCCATCGTGAAT

7710_donor CCATAACCCTTAATGTCTATTTATATACATAGACTCCTTCCTAATCCGCCCATCGTGAAT

M32_outgroup CCATAACCCTTAATGTCTATTTATATACATAGACTCCTTCCTAATCCGCCCATCGTGAAT

7710_outgroup CCATAACCCTTAATGTCTATTTATATACATAGACTCCTTCCTA-TCCGCCCATCGTGAAT

******************************************* ****************

IAA_insertion AAGGAAAGGCCCATCGGTATCTACACAAATAAAAAACTGATCCCACACTCTATAAAAGAC

M32_donor AAGGAAAGGCCCATCGGTATCTACACAAATAAAAAACTGATCCCACACTCTATAAAAGAC

7710_donor AAGGAAAGGCCCATCGGTATCTACACAAATAAAAAACTGATCCCACACTCTATAAAAGAC

M32_outgroup AAGGAAAGGCCCATCGGTATCTACACAAATAAAAAACTGATCCCACACTCTATAAAAGAC

7710_outgroup AAGGAAAGGCCCATCG-TATCTAC-CAAATAAAAA-CTGATCCCACACTCTATAAAAGAC

**************** ******* ********** ************************

IAA_insertion GTAAATAGGCCCAATAATAATTACTTAATTGGATCACTTTAGTTTTGGGCCACACCGTAT

M32_donor GTAAATAGGCCCAATAATAATTACTTAATTGGATCACTTTAGTTTTGGGCCACACCGTAT

7710_donor GTAAATAGGCCCAATAATAATTACTTAATTGGATCACTTTAGTTTTGGGCCACACCGTAT

M32_outgroup GTAAATAGGCCCAATAATAATTACTTAATTGGATCACTTTAGTTTTGGGCCACACCGTAT

7710_outgroup GTAAATAGGCCCAATA-TAATTACTTAATTGGATCACTTTAGTTTTGGGCCACACCGTAT

**************** *******************************************

IAA_insertion GATAGCACATAATACAATTATAACTGAATTGCACACGTATTTGTATTTAGGTCCATAAAA

M32_donor GATAGCACATAATACAATTATAACTGAATTGCACACGTATTTGTATTTAGGTCCATAAAA

7710_donor GATAGCACATAATACAATTATAACTGAATTGCACACGTATTTGTATTTAGGTCCATAAAA

M32_outgroup GATAGCACATAATACAATTATAACTGAATTGCACACGTATTTGTATTTAGGTCCATAAAA

7710_outgroup GATAGCACA--ATACAATTATAACTGAATTGCACACGA--TTGTATTTAGGTCCATAAAA

********* ************************** ********************

IAA_insertion TTTCCAACAGTCTCCCACTTGGACCAAATACAATTACAACGTGTGTGATACTTGATAATT

M32_donor TTTCCAACAGTCTCCCACTTGGACCAAATACAATTACAACGTGTGTGATACTTGATAATT

7710_donor TTTCCAACAGTCTCCCACTTGGACCAAATACAATTACAACGTGTGTGATACTTGATAATT

M32_outgroup TTTCCAACAGTCTCCCACTTGGACCAAATACAATTACAACGTGTGTGATACTTGATAATT

7710_outgroup TTTCCAACAGTCTCCCACTTGGACCAAATACAATTACAACGTGTGTGATACTTGATAATT

************************************************************

IAA_insertion GCATTATACTTTATAA-CCTTATGAGCTCAAAATTGCTATCAAAATCTCAACGTCTTTAA

M32_donor GCATTATACTTTATAA-CCTTATGAGCTCAAAATTGCTATCAAAATCTCAACGTCTTTAA

7710_donor GCATTATACTTTATAA-CCTTATGAGCTCAAAATTGCTATCAAAATCTCAACGTCTTTAA

M32_outgroup GCATTATACTTTATAA-CCTTATGAGCTCAAAATTGCTATCAAAATCTCAACGTCTTTAA

7710_outgroup GCATTATACTTTATAAACCTTATGAGCTCAAA-TTGCTATCAAAATCTCAACGTCTTTAA

**************** *************** ***************************

IAA_insertion ACAATTCAGTCCATTAATTACATCAACACAGGATCAAAGAGATCTATGCTACATTTGCCG

M32_donor ACAATTCAGTCCATTAATTACATCAACACAGGATCAAAGAGATCTATGCTACATTTGCCG

7710_donor ACAATTCAGTCCATTAATTACATCAACACAGGATCAAAGAGATCTATGCTACATTTGCCG

M32_outgroup ACAATTCAGTCCATTAATTACATCAACACAGGATCAAAGAGATCTATGCTACATTTGCCG

7710_outgroup ACAATTCAGTCCATTAATTACC---ACACAGGATCAAAGAGATCTATGCTACATTTGCCG

********************* ***********************************

IAA_insertion TAACCAGACCCATCAATGGTCACAATGTCAACATAATTAACGACATGAATCAAGCATGGG

M32_donor TAACCAGACCCATCAATGGTCACAATGTCAACATAATTAACGACATGAATCAAGCATGGG

7710_donor TAACCAGACCCATCAATGGTCACAATGTCAACATAATTAACGACATGAATCAAGCATGGG

M32_outgroup TAACCAGACCCATCAATGGTCACAATGTCAACATAATTAACGACATGAATCAAGCATGGG

7710_outgroup TAACCAGACCCATCAATGGTCACAATGTCAACATAATTAACGACATGAATCA-GCATGGG

**************************************************** *******

IAA_insertion TGTGTAGCATGGAAATTACATACAAATGTGATCCAAGTATGCCTATTTCCAACTGGTCCA

M32_donor TGTGTAGCATGGAAATTACATACAAATGTGATCCAAGTATGCCTATTTCCAACTGGTCCA

7710_donor TGTGTAGCATGGAAATTACATACAAATGTGATCCAAGTATGCCTATTTCCAACTGGTCCA

M32_outgroup TGTGTAGCATGGAAATTACATACAAATGTGATCCAAGTATGCCTATTTCCAACTGGTCCA

7710_outgroup TGTGTAGCATGGAAATTACATACAAATGTGATCCAAGTATGCCT-TTTCCAACTGGTCCA

******************************************** ***************

IAA_insertion CTGTAAACTTTGGTAAGATCAACAATATGATCTAATCGAAGAGTAAACCGAACCGAATAC

M32_donor CTGTAAACTTTGGTAAGATCAACAATATGATCTAATCGAAGAGTAAACCGAACCGAATAC

7710_donor CTGTAAACTTTGGTAAGATCAACAATATGATCTAATCGAAGAGTAAACCGAACCGAATAC

M32_outgroup CTGTAAACTTTGGTAAGATCAACAATATGATCTAATCGAAGAGTAAACCGAACCGAATAC

7710_outgroup CTGTAAACTTTGGTAAGATCAACAATATGATCTAATCGAAGAGTAA-CCGAACCGAATAC

********************************************** *************

IAA_insertion CTTATTTCTGCAGAAAATACCTTAAACCTTAATATCCGAATTAGAACTTCATCATACAAG

M32_donor CTTATTTCTGCAGAAAATACCTTAAACCTTAATATCCGAATTAGAACTTCATCATACAAG

7710_donor CTTATTTCTGCAGAAAATACCTTAAACCTTAATATCCGAATTAGAACTTCATCATACAAG

M32_outgroup CTTATTTCTGCAGAAAATACCTTAAACCTTAATATCCGAATTAGAACTTCATCATACAAG

7710_outgroup CTTATTTCTGCAGAAA-TACCTTAAACCTTAATATCCGAATTAGAACTTCATCATACAAG

**************** *******************************************

IAA_insertion CATCAACATAACAAACTCCCACTGAAACTGTATATCCTTAATACCTTAACTGGCATGACA

M32_donor CATCAACATAACAAACTCCCACTGAAACTGTATATCCTTAATACCTTAACTGGCATGACA

7710_donor CATCAACATAACAAACTCCCACTGAAACTGTATATCCTTAATACCTTAACTGGCATGACA

M32_outgroup CATCAACATAACAAACTCCCACTGAAACTGTATATCCTTAATACCTTAACTGGCATGACA

7710_outgroup CATCAACATAACAAACTCCCACTGAAACTGTATATCCTTAATACCTTAACTGGCATGACA

************************************************************

IAA_insertion CACATGAATAATGTGCTCATGAAATACTTTAAGTATTTAACTTTAGTAAGCGGATCCGCA

M32_donor CACATGAATAATGTGCTCATGAAATACTTTAAGTATTTAACTTTAGTAAGCGGATCCGCA

7710_donor CACATGAATAATGTGCTCATGAAATACTTTAAGTATTTAACTTTAGTAAGCGGATCCGCA

M32_outgroup CACATGAATAATGTGCTCATGAAATACTTTAAGTATTTAACTTTAGTAAGCGGATCCGCA

7710_outgroup CACATGAATAATGTGCTCATGAAATACTTTAAGTATTTAACTTTAGTAAGCGGATCCGCA

************************************************************

IAA_insertion ATAATGGAGTTGTATTATGATGCTTTAAGTACAATAATTTTACTCTTCAAACTTTCACAA

M32_donor ATAATGGAGTTGTATTATGATGCTTTAAGTACAATAATTTTACTCTTCAAACTTTCACAA

7710_donor ATAATGGAGTTGTATTATGATGCTTTAAGTACAATAATTTTACTCTTCAAACTTTCACAA

M32_outgroup ATAATGGAGTTGTATTATGATGCTTTAAGTACAATAATTTTACTCTTCAAACTTTCACAA

7710_outgroup ATAATGGAGTTGTATTATGATGCTTTAAGTACAATAATTTTACTCTTCAAACTTTCACA-

***********************************************************

IAA_insertion CACTAAAGCCATGATAAAAATTTAACCGTATTTGGATTTCAAGCTACCTTGACATCCACC

M32_donor CACTAAAGCCATGATAAAAATTTAACCGTATTTGGATTTCAAGCTACCTTGACATCCACC

7710_donor CACTAAAGCCATGATAAAAATTTAACCGTATTTGGATTTCAAGCTACCTTGACATCCACC

M32_outgroup CACTAAAGCCATGATAAAAATTTAACCGTATTTGGATTTCAAGCTACCTTGACATCCACC

7710_outgroup CACTAAAGCCATGATAAAAATTTAACCGTATTTGGATTTCAAGCTACCTTGACATCCACC

************************************************************

IAA_insertion AAAAACGGAGTCTAATAGCTGTAAGTTCTCTCTAATGGATCTCTTTAATGATACTTATTC

M32_donor AAAAACGGAGTCTAATAGCTGTAAGTTCTCTCTAATGGATCTCTTTAATGATACTTATTC

7710_donor AAAAACGGAGTCTAATAGCTGTAAGTTCTCTCTAATGGATCTCTTTAATGATACTTATTC

M32_outgroup AAAAACGGAGTCTAATAGCTGTAAGTTCTCTCTAATGGATCTCTTTAATGATACTTATTC

7710_outgroup AAAA-CGGAGTCTAATAGCTGTAAGTTCTCTCTAATG-ATCTCTTTAATGATACTTATTC

**** ******************************** **********************

IAA_insertion TTGAGGTTGTTGAGTTTGCTCATTGGAATTTTCCTTTTGAGATCCTAAATAGGGGAATAA

M32_donor TTGAGGTTGTTGAGTTTGCTCATTGGAATTTTCCTTTTGAGATCCTAAATAGGGGAATAA

7710_donor TTGAGGTTGTTGAGTTTGCTCATTGGAATTTTCCTTTTGAGATCCTAAATAGGGGAATAA

M32_outgroup TTGAGGTTGTTGAGTTTGCTCATTGGAATTTTCCTTTTGAGATCCTAAATAGGGGAATAA

7710_outgroup TTGAGGTTGTTGAGTTTGCTCATTGGAATTTTCCTTTTGAGATCCTAAATAGGGGAATAA

************************************************************

IAA_insertion ATCCTTATCTTCCCCCAAACTCAACATCCTCAAAGGACAAGCATCTATTGTATCCAAAAA

M32_donor ATCCTTATCTTCCCCCAAACTCAACATCCTCAAAGGACAAGCATCTATTGTATCCAAAAA

7710_donor ATCCTTATCTTCCCCCAAACTCAACATCCTCAAAGGACAAGCATCTATTGTATCCAAAAA

M32_outgroup ATCCTTATCTTCCCCCAAACTCAACATCCTCAAAGGACAAGCATCTATTGTATCCAAAAA

7710_outgroup ATCCTTATCTTCCCCCAAACTCAACATCCTCAAAGGACAAGCATCTATTGTATCCAAAAA

************************************************************

IAA_insertion TTGATCCAATAGTGAGACTATAAAAAAAAATGTATCCTTTATTATTATCTTCATCAATAT

M32_donor TTGATCCAATAGTGAGACTATAAAAAAAA-TGTATCCTTTATTATTATCTTCATCAATAT

7710_donor TTGATCCAATAGTGAGACTATAAAAAAAA-TGTATCCTTTATTATTATCTTCATCAATAT

M32_outgroup TTGATCCAATAGTGAGACTATAAAAAAAA-TGTATCCTTTATCATTATCTTCATCAATAT

7710_outgroup TTGATCCAATAGTGAGACTATAAAAAAAA-TGTATCCTTTATCATTATCTTCATCAATAT

***************************** ************ *****************

IAA_insertion CTTTAGTGAAAGATGTCAAGTGAGCACTTTATATCTTTCATTGTTTCAATCTCTCTTCCT

M32_donor CTTTAGTGAAAGATGTCAAGTGAGCACTTTATATCTTTCATTGTTTCAATCTCTCTTCCT

7710_donor CTTTAGTGAAAGATGTCAAGTGAGCACTTTATATCTTTCATTGTTTCAATCTCTCTTCCT

M32_outgroup CTTTAGTGAAAGATGTCAAGTGAGCACTTTATATCTTTCATTGTTTCAATCTCTCTTCCT

7710_outgroup CTTTAGTGAAAGATGTCAAGTGAGCACTTTATATCTTTCATTGTTTCAATCTCTCTTCCT

************************************************************

IAA_insertion CCGAATCAACATGAGTAGTGAACTCATTTAGAGGTTCACTTCTCCATAACAACAATTTAG

M32_donor CCGAATCAACATGAGTAGTGAACTCATTTAGAGGTTCACTTCTCCATAACAACAATTTAG

7710_donor CCGAATCAACATGAGTAGTGAACTCATTTAGAGGTTCACTTCTCCATAACAACAATTTAG

M32_outgroup CCGAATCAACATGAGTAGTGAACTCATTTAGAGGTTCACTTCTCCATAACAACAATTTAG

7710_outgroup CCGAATCAACATGAGTAGTGAACTCATTTAGAG-TTCACTTCTCCATAACAA---TTTAG

********************************* ****************** *****

IAA_insertion GAAGAGATTTAAAAACAAATGCACAAGCAAGTCGTTAGACTCCTCCAATTTAAGTGCCCT

M32_donor GAAGAGATTTAAAAACAAATGCACAAGCAAGTCGTTAGACTCCTCCAATTTAAGTGCCCT

7710_donor GAAGAGATTTAAAAACAAATGCACAAGCAAGTCGTTAGACTCCTCCAATTTAAGTGCCCT

M32_outgroup GAAGAGATTTAAAAACAAATGCACAAGCAAGTCGTTAGACTCCTCCAATTTAAGTGCCCT

7710_outgroup GAAGAGATTTAAAA-CAAATGCACAAGCAAGTCGTTAGACTCCTCCAATTTAAGTGCCCT

************** *********************************************

IAA_insertion TAAATCATGAAACAATATCACAAATTTCCATAATGTGCTCCCTTAATTTATTTATTTTCC

M32_donor TAAATCATGAAACAATATCACAAATTTCCATAATGTGCTCCCTTAATTTATTTATTTTCC

7710_donor TAAATCATGAAACAATATCACAAATTTCCATAATGTGCTCCCTTAATTTATTTATTTTCC

M32_outgroup TAAATCATGAAACAATATCACAAATTTCCATAATGTGCTCCCTTAATTTATTTATTTTCC

7710_outgroup TAA-TCATGAAACAATATCACAAATT-CCATAATGTGCTCCCTTAATTTATTTATTTTCC

*** ********************** *********************************

IAA_insertion CCTTAATACCTTTTTCCCTTATGGAATTTAAGTCCAACAAGGTTGTACTCAATTCCCCTG

M32_donor CCTTAATACCTTTTTCCCTTATGGAATTTAAGTCCAACAAGGTTGTACTCAATTCCCCTG

7710_donor CCTTAATACCTTTTTCCCTTATGGAATTTAAGTCCAACAAGGTTGTACTCAATTCCCCTG

M32_outgroup CCTTAATACCTTTTTCCCTTATGGAATTTAAGTCCAACAAGGTTGTACTCAATTCCCCTA

7710_outgroup CCTTAATACCTTTTTCCCTTATGGAATTTAAGTCCAACAAGGTTGTACTCA-TTCCCCTA

*************************************************** *******

IAA_insertion ATCTTTATAGCAAAACACTTTAAGCTCTTTAGAGAACAATTTGACATCTTTCTCTTTCAT

M32_donor ATCTTTATAGCAAAACACTTTAAGCTCTTTAGAGAACAATTTGACATCTTTCTCTTTCAT

7710_donor ATCTTTATAGCAAAACACTTTAAGCTCTTTAGAGAACAATTTGACATCTTTCTCTTTCAT

M32_outgroup ATCTTTATAGCAAAACACTTTAAGCTCTTTAGAGAACAATTTGACATCTTTCTCTTTCAT

7710_outgroup ATCTTTATAGCAAA-CACTTTAAGCTCTTTAGAGAACAATTTGACATCTTTCTCTTTCAT

************** *********************************************

IAA_insertion TTGGACATACATAATGTCCCCTAATAACTCCGAAATTGTACTTTAAATTGCACTTTACAT

M32_donor TTGGACATACATAATGTCCCCTAATAACTCCGAAATTGTACTTTAAATTGCACTTTACAT

7710_donor TTGGACATACATAATGTCCCCTAATAACTCCGAAATTGTACTTTAAATTGCACTTTACAT

M32_outgroup TTGGACATACATAATGTCCCCTAATAACTCCGAAATTGTACTTTAAATTGCACTTTACAT

7710_outgroup TTGGACATACATAATGTCCCCTAATAACTCCGAAATTGTACTTTAAATTGCACTTTACAT

************************************************************

IAA_insertion GATCATATACGACCTATCCAATTTGAGCATTCTCACTTCTCCATATTCCCCTTTAGTAGG

M32_donor GATCATATACGACCTATCCAATTTGAGCATTCTCACTTCTCCATATTCCCCTTTAGTAGG

7710_donor GATCATATACGACCTATCCAATTTGAGCATTCTCACTTCTCCATATTCCCCTTTAGTAGG

M32_outgroup GATCATATACGACCTATGCAATTTGAGCATTCTCACTTCTCCATATTCCCCTTTAGTAGG

7710_outgroup GATCATATACG-CCTATGCAATTTGAGCATTCTC-CTTCTCCATATTCCCCTTTAGTAGG

*********** ***** **************** *************************

IAA_insertion AGCACTAAAATCCATAAGAGTTGCGAGTGCTCAACTCGAAAAGCAAGGTCTGATCTAGAT

M32_donor AGCACTAAAATCCATAAGAGTTGCGAGTGCTCAACTCGAAAAGCAAGGTCTGATCTAGAT

7710_donor AGCACTAAAATCCATAAGAGTTGCGAGTGCTCAACTCGAAAAGCAAGGTCTGATCTGGAT

M32_outgroup AGCACTAAAATCCGTAAGAGTTGCGAGTGCTCAACTCGAAAAGCAAGGTCTGATCTAGAT

7710_outgroup --CACTAAAATCCGTAAGAGTTGCGAGTGCTCAACTCGAAAAGCAAGGTCTGATCTAGAT

*********** ****************************************** ***

IAA_insertion CCATGCAAAAAAAAAAAAAAAA-CCAAAACCTTCAATTTATTATCCTTAATCAGATTAAT

M32_donor CCATGCAAAAAAAAAAAAAAA--CCAAAACCTTCAATTTATTATCCTTAATCAGATTAAT

7710_donor CCATGCAAAAAAAAAAAAAAAAACCAAAACCTTCAATTTATTATCCTTAATCAGATTAAT

M32_outgroup CCATGCAAAAAAAAAAAAAA---CCAAAACCTTCAATTTATTATCCTTAATCAGATTAAT

7710_outgroup CCATGCAAAAAAAAAAAA-----CCAAAACCTTCA-TTTATTATCCTTAATCAGATTAAT

****************** ************ ************************

IAA_insertion ATATTGACAATGTATCCCATCATGTACAAGAGACCTAACACAACATTAATTTTTT-AGTC

M32_donor ATATTGACAATGTATCCCATCATGTACAAGAGACCTAACACAACATTAATTTTTT-AGTC

7710_donor ATATTGACAATGTATCCCATCATGTACAAGAGACCTAACACAACATTAATTTTTT-AGTC

M32_outgroup ATATTGACAATGTATCCCATCATGTACAAGAGACCTAACACAACATTAATTTTTTTAGTC

7710_outgroup ATATTGACAATGTATCCCATCATGTACAAGAGACCTAACACAACATTAATTTTTTTAGTC

******************************************************* ****

IAA_insertion TTTGGACAGAAAAATTAACTTGTAAGTGGTCCTCTTGGTGTAGTAATCAAATACTAACAA

M32_donor TTTGGACAGAAAAATTAACTTGTAAGTGGTCCTCTTGGTGTAGTAATCAAATACTAACAA

7710_donor TTTGGACAGAAAAATTAACTTGTAAGTGGTCCTCTTGGTGTAGTAATCAAATACTAACAA

M32_outgroup TTTGGACAGACAAATTAACTTGTAAGTGGTCCTCTTGGTGTAGTAATCAAATACTAACAA

7710_outgroup TTTGGACAGACAAATTAACTTGTAAGTGGTCCTCTTGGTGTAGTAATCAAATACTAACAA

********** *************************************************

IAA_insertion TAAATTCATGTCAATGTTAAGCCTTTCTTTGGACCGACTTAACAACGCATGAAAAACCGA

M32_donor TAAATTCATGTCAATGTTAAGCCTTTCTTTGGACCGACTTAACAACGCATGAAAAACCGA

7710_donor TAAATTCATGTCAATGTTAAGCCTTTCTTTGGACCGACTTAACAACGCATGAAAAACCGA

M32_outgroup TAAATTCATGTCAATGTTAAGCCTTTCTTTGGATCGACTTAACAACGCATGAAAAACCGA

7710_outgroup TAAATTCATGTCAATGTTAAGCCTTTCTTTGGATCGACTTAAC---GCATGAAAAACCGA

********************************* ********* **************

IAA_insertion ACAATTGTTACATATTTATTACCACAAGCATGTTTAATTTCCGGCCAAATAATAGCCTTC

M32_donor ACAATTGTTACATATTTATTACCACAAGCATGTTTAATTTCCGGCCAAATAATAGCCTTC

7710_donor ACAATTGTTACATATTTATTACCACAAGCATGTTTAATTTCCGGCCAAATAATAGCCTTC

M32_outgroup ACAATTGTTACATATTTATTACCACAAGCATGTTTAATTTCCGGCCAAATAATAGCCTTC

7710_outgroup ACAATTGTTACATATTTATTACCACAAGCATGTTTAATTTCCGGCCAAATAATAGCCTTC

************************************************************

IAA_insertion CTTTGGGCCGACCATTATCCGCATGAAAACTAAACACACACAGGTATCTCAATATTTCAA

M32_donor CTTTGGGCCGACCATTATCCGCATGAAAACTAAACACACACAGGTATCTCAATATTTCAA

7710_donor CTTTGGGCCGACCATTATCCGCATGAAAACTAAACACACACAGGTATCTCAATATTTCAA

M32_outgroup CTTTGGGCCGACCATTATCCGCATGAAAACTAAACACACACAGGTATCTCAATATTTCAA

7710_outgroup CTTTGGGCCGACCATTATCCGCATGAAAACTAAACACACACAGGTATCTCAATATTTCAA

************************************************************

IAA_insertion ATTAATTGATCTACACAAAAGAGGTTACTTTGGCAACATATTGTTTCAATCAATTAATCC

M32_donor ATTAATTGATCTACACAAAAGAGGTTACTTTGGCAACATATTGTTTCAATCAATTAATCC

7710_donor ATTAATTGATCTACACAAAAGAGGTTACTTTGGCAACATATTGTTTCAATCAATTAATCC

M32_outgroup ATTAATTGATCTACACAAAAGAGGTTACTTTGGCAACATATTGTTTCAATCAATTAATCC

7710_outgroup -TTAATTGATCTACACAAAAGAG--TACTTTGGCAACATAT-GTTTCAATCAATTAATCC

********************** **************** ******************

IAA_insertion AAAATACTAGACCCAACTTAATAACCAAATTGCCAATTATTACAATCCGAAATATATGAT

M32_donor AAAATACTAGACCCAACTTAATAACCAAATTGCCAATTATTACAATCCGAAATATATGAT

7710_donor AAAATACTAGACCCAACTTAATAACCAAATTGCCAATTATTACAATCCGAAATATATGAT

M32_outgroup AAAATACTAGACCCAACTTAATAACCAAATTGCCAATTATTACAATCCGAAATATATGAT

7710_outgroup AAAATACTAGACCCAACTTAATAACCAAATTGCCAAT-ATTACAATCCGAA-TATATGA-

************************************* ************* *******

IAA_insertion GAATCCATAATTAGTCATGTGTTTATGAATCCATGACCTCAAATAGATAAGATGAAGCGC

M32_donor GAATCCATAATTAGTCATGTGTTTATGAATCCATGACCTCAAATAGATAAGATGAAGCGC

7710_donor GAATCCATAATTAGTCATGTGTTTATGAATCCATGACCTCAAATAGATAAGATGAAGCGC

M32_outgroup GAATCCATAATTAGTCATGTGTTTATGAATCCATGACCTCAAATAGATAAGATGAAGCGC

7710_outgroup --ATCCATAATTAGTCATGTGTTTATGAATCCATGACCTCAA-TAGATAAGATGAAGCGC

**************************************** *****************

IAA_insertion AAAATAAGTTGTAAACTGTAACACTAAAAATTAATCGATGGATTCATAATTCATAGCAAC

M32_donor AAAATAAGTTGTAAACTGTAACACTAAAAATTAATCGATGGATTCATAATTCATAGCAAC

7710_donor AAAATAAGTTGTAAACTGTAACACTAAAAATTAATCGATGGATTCATAATTCATAGCAAC

M32_outgroup AAAATAAGTTGTAAACTGTAACACTAAAAATTAATCGATGGATTCATAATTCATAGCAAC

7710_outgroup AAA-TAAGTTGTAAACTGTAACACTAAAA-TTAATCGCTGGATTCATAATTCATAGCACA

*** ************************* ******* ********************

IAA_insertion ACAGCAGCGACTAACAATTGAATTAAGCAATAAATTGTTCTGTCTTATATATGGAGTAAG

M32_donor ACAGCAGCGACTAACAATTGAATTAAGCAATAAATTGTTCTGTCTTATATATGGAGTAAG

7710_donor ACAGCAGCGACTAACAATTGAATTAAGCAATAAATTGTTCTGTCTTATATATGGAGTAAG

M32_outgroup ACAGCAGCGACTAACAATTGAATTAAGCAATAAATTGTTCTGTCTTATATATGGAGTAAG

7710_outgroup CCAGCA-CGACTAACAATTGAATTAAGCAATAAATT--TCTGTCTTATATATGGAGTAAG

***** ***************************** **********************

IAA_insertion ATTAAAATACCAAAGACTTTAACC--ACCAACACCCAGGCCAGGTCAAAAAATCAACCGA

M32_donor ATTAAAATACCAAAGACTTTAACC--ACCAACACCCAGGCCAGGTCAAAAAATCAACCGA

7710_donor ATTAAAATACCAAAGACTTTAACC--ACCAACACCCAGGCCAGGTCAAAAAATCAACCGA

M32_outgroup ATTAAAATACCAAAGACTTTAACC--ACCAACACCCAGGCCAGGTCAAAAAATCAACCGA

7710_outgroup ATTAAAA--CCAAAGACTTTAACCCCACCAACACCCAGGCCAGGTCAAAAAATCAACCGA

******* *************** **********************************

IAA_insertion AATAAATAAAGTCAAGCTACATATATCTAGTGACTTTATCGATTCTAATCTTAAGAAAGG

M32_donor AATAAATAAAGTCAAGCTACATATATCTAGTGACTTTATCGATTCTAATCTTAAGAAAGG

7710_donor AATAAATAAAGTCAAGCTACATATATCTAGTGACTTTATCGATTCTAATCTTAAGAAAGG

M32_outgroup AATAAATAAAGTCAAGCTACATATATCTAGTGACTTTATCGATTCTAATCTTAAGAAAGG

7710_outgroup AATAAATAA-GTCAAGCTACATATATCTAGTGACTTTATCGATTCTAATCTTAAGAAAGG

********* **************************************************

IAA_insertion AATCACCAAATCAATGCCAAAACTAAAAAATAATCAGAACGATGACCAACCATATTTGAA

M32_donor AATCACCAAATCAATGCCAAAACTAAAAAATAATCAGAACGATGACCAACCATATTTGAA

7710_donor AATCACCAAATCAATGCCAAAACTAAAAAATAATCAGAACGATGACCAACCATATTTGAA

M32_outgroup AATCACCAAATCAATGCCAAAACTAAAAAATAATCAGAACGATGACCAACCATATTTGAA

7710_outgroup AATCACCAAATCAATGCCAAAACTAAAAAATAATCAGAACGATGACCA-CCATATTTGAA

************************************************ ***********

IAA_insertion GCACCCACAAAGCCTCATATGTCGAAATAAAATATCTGAAGTAAACTGTAAGGGATATTC

M32_donor GCACCCACAAAGCCTCATATGTCGAAATAAAATATCTGAAGTAAACTGTAAGGGATATTC

7710_donor GCACCCACAAAGCCTCATATGTCGAAATAAAATATCTGAAGTAAACTGTAAGGGATATTC

M32_outgroup GCACCCACAAAGCCTCATATGTCGAAATAAAATATCTGAAGTAAACTGTAAGGGATATTC

7710_outgroup GCACCCACAAAGCCTCATATGTCGAAATAAAATATCTGAAGTAAACTGTAAGGGATATTC

************************************************************

IAA_insertion AATTAAAGCATAATAGGGAATCAAAACCCAAATTAAATTTGGATGCTAACAAAGCCAAAA

M32_donor AATTAAAGCATAATAGGGAATCAAAACCCAAATTAAATTTGGATGCTAACAAAGCCAAAA

7710_donor AATTAAAGCATAATAGGGAATCAAAACCCAAATTAAATTTGGATGCTAACAAAGCCAAAA

M32_outgroup AATTAAAGCATAATAGGGAATCAAAACCCAAATTAAATTTGGATGCTAACAAAGCCAAAA

7710_outgroup AATTAA-GCATAATAGGGAATCAAAACCCAAATTAAATTTGGATGCTAACAAAGCCAAAA

****** *****************************************************

IAA_insertion ACATACACACACAACTGTCAACTGGATCGAATTGTGCATGTCAAATTGTCAATCATGACA

M32_donor ACATACACACACAACTGTCAACTGGATCGAATTGTGCATGTCAAATTGTCAATCATGACA

7710_donor ACATACACACACAACTGTCAACTGGATCGAATTGTGCATGTCAAATTGTCAATCATGACA

M32_outgroup ACATACACACACAACTGTCAACTGGATCGAATTGTGCATGTCAAATTGTCAATCATGACA

7710_outgroup ACATACACACACAACTGTCAACTGGATCGA-TTGTGCATGTCAA-TTGTCAATCATGACA

****************************** ************* ***************

IAA_insertion ATTGAGAAATAATTGATAACATACATCTGCCTTTTAGGCCTCGTCAATTAACAAATACTC

M32_donor ATTGAGAAATAATTGATAACATACATCTGCCTTTTAGGCCTCGTCAATTAACAAATACTC

7710_donor ATTGAGAAATAATTGATAACATACATCTGCCTTTTAGGCCTCGTCAATTAACAAATACTC

M32_outgroup ATTGAGAAATAATTGATAACATACATCTGCCTTTTAGGCCTCGTCAATTAACAAATACTC

7710_outgroup ATTGAGAAATAATTGATAACATACATCTGCCTTTTAGGCCTCGTCAATAAC--AATACTC

************************************************ * *******

IAA_insertion AAACTCAATAGTTGATCACAAAACAAATTCGAAGTTCTGAAAGAAAAATACTTCGTACCA

M32_donor AAACTCAATAGTTGATCACAAAACAAATTCGAAGTTCTGAAAGAAAAATACTTCGTACCA

7710_donor AAACTCAATAGTTGATCACAAAACAAATTCGAAGTTCTGAAAGAAAAATACTTCGTACCA

M32_outgroup AAACTCAATAGTTGATCACAAAACAAATTCGAAGTTCTGAAAGAAAAATACTTCGTACCA

7710_outgroup AAACTCAATAGTTGATCACAAAACAAATTCGA-GTTCTGAAAGAAAAATACTTCGTACCA

******************************** ***************************

IAA_insertion AATAATTTAATATGAAGAAGGAGATCCATAAAGAATTGGACTTGTTGGGATTAAACCCTA

M32_donor AATAATTTAATATGAAGAAGGAGATCCATAAAGAATTGGACTTGTTGGGATTAAACCCTA

7710_donor AATAATTTAATATGAAGAAGGAGATCCATAAAGAATTGGACTTGTTGGGATTAAACCCTA

M32_outgroup AATAATTTAATATGAAGAAGGAGATCCATAAAGAATTGGACTAGTTGGGATTAAACCCTA

7710_outgroup A-TAATTTAATATGAAGAAGGAGATCCATAAAGAATTGGACTAGTTGGGATTAA-CCCTA

* **************************************** *********** *****

IAA_insertion ATTCATATCAAATTAGTCGGTAATAATAGATCTGCAGAAGCATACCTGAATCCATGACGA

M32_donor ATTCATATCAAATTAGTCGGTAATAATAGATCTGCAGAAGCATACCTGAATCCATGACGA

7710_donor ATTCATATCAAATTAGTCGGTAATAATAGATCTGCAGAAGCGTACCTGAATCCATGACGA

M32_outgroup ATTCATATCAAATTAGTCGGTAATAATAGATCTGCAGAAGCATACCTGAATCCATGACGA

7710_outgroup ATTCATATCAAATTAGTCG-TAATAATAGATCTGCAGAAGCATACCTGAATCCATGACGA

******************* ********************* ******************

IAA_insertion AGATCGGCGGTGGGTTTTGATCTTCCAATTCTTTATGGCTCCTTAGGGTTTCTACTGATG

M32_donor AGATCGGCGGTGGGTTTTGATCTTCCAATTCTTTATGGCTCCTTAGGGTTTCTACTGATG

7710_donor AGATCGGCGGTGGGTTTTGATCTTCCAATTCTTTATGGCTCCTTAGGGTTTCTACTGATG

M32_outgroup AGATCGGCGGTGGGTTTTGATCTTCCAATTCTTTATGGCTCCTTAGGGTTTCTACTGATG

7710_outgroup AGATCGGCGG--GGTTTTGATCTTCCAATTCTTTATGGCTCCTTAGG-TTTCTACTGATG

********** *********************************** ************

IAA_insertion ATGGGATGTCAGTGAGAATAGGAAAACCATATGAATCGGGGACCATAACCCTTAATGTCT

M32_donor ATGGGATGTCAGTGAGAATAGGAAAACCATATGAATCGGGGACCATAACCCTTAATGTCT

7710_donor ATGGGATGTCAGTGAGAATAGGAAAACCATATGAATCGGGGACCATAACCCTTAATGTCT

M32_outgroup ATGGGATGTCAGTGAGAATAGGAAAACCATATAAATCGGGGACCATAACCCTTAATGTCT

7710_outgroup --GGGATGTCAGTGAGAATAGGAAAACCATATAAATCGGGGACCATAACCCTTAATGTCT

****************************** ***************************

IAA_insertion ATTTATATACATAGACTCCTTCCTAATCCGCCCATCGTGAATAAGGAAAGGCCCATCGGT

M32_donor ATTTATATACATAGACTCCTTCCTAATCCGCCCATCGTGAATAAGGAAAGGCCCATCGGT

7710_donor ATTTATATACATAGACTCCTTCCTAATCCGCCCATCGTGAATAAGGAAAGGCCCATCGGT

M32_outgroup ATTTATATACATAGACTCCTTCCTAATCCGCCCATCGTGAATAAGGAAAGGCCCATCGGT

7710_outgroup ATTTATATACATAGACTCCTTCCTAATCC-CCCATCGTGAATAAGGAAAGGCCCATCG-T

***************************** **************************** *

IAA_insertion ATCTACACAAATAAAAAACTGATCCCACACTCTATAAAAGACGTAAATAGGCCCAATAAT

M32_donor ATCTACACAAATAAAAAACTGATCCCACACTCTATAAAAGACGTAAATAGGCCCAATAAT

7710_donor ATCTACACAAATAAAAAACTGATCCCACACTCTATAAAAGACGTAAATAGGCCCAATAAT

M32_outgroup ATCTACACAAATAAAAAACTGATCCCACACTCTATAAAAGACGTAAATAGGCCCAATAAT

7710_outgroup ATCTACACAAATAAAAAACTGATCCCACACTCTATAAAA--CGTAAATAG-CCCAATAAT

*************************************** ********* *********

IAA_insertion AATTACTTAATTGGATCACTTTAGTTTTGGGCCACACCGTATGATAGCACATAATACAAT

M32_donor AATTACTTAATTGGATCACTTTAGTTTTGGGCCACACCGTATGATAGCACATAATACAAT

7710_donor AATTACTTAATTGGATCACTTTAGTTTTGGGCCACACCGTATGATAGCACATAATACAAT

M32_outgroup AATTACTTAATTGGATCACTTTAGTTTTGGGCCACACCGTATGATAGCACATAATACAAT

7710_outgroup AATTACTTAATTGGATCACTTTAGTTTTGGGCCACACCGTATGATAGCACATAATACAAT

************************************************************

IAA_insertion TATAACTGAATTGCACACGTATTTGTATTTAGGTCCATAAAATTTCCAACATGGCC

M32_donor TATAACTGAATTGCACACGTATTTGTATTTAGGTCCATAAAATTTCCAACA-----

7710_donor TATAACTGAATTGCACACGTATTTGTATTTAGGTCCATAAAATTTCCAACA-----

M32_outgroup TATAACTGAATTGCACACGTATTTGTATTTAGGTCCATAAAATTTCCAACA-----

7710_outgroup TATAACTGAATTGCACACGTATT-GTATTTAGGTCCATAAAATTTCCAACA-----

*********************** ***************************

Table S7. Parameter estimates and their standard errors for three-parameter exponential decay models predicting the binding intensity of mutant (Mut) and wildtype (WT) degron peptides measured during the wash phase of a surface plasmon resonance experiment. Binding intensity measured between TIR1 and degron peptides in the presence of one of several auxin analogs or DMSO as a negative control. The equation fitted for each peptide/auxin combination was $y_{t}=c+\left( d-c \right)e^{-t/\propto}$ where *y_t_* is the predicted binding intensity at time t, *c* is the lower limit of binding intensity as t approaches infinity, *d* is the upper limit of binding intensity at the beginning of the experiment, and $\propto$ is the rate of decay. Smaller values of $\propto$ indicate faster decay. DMSO did not fit to the model due to lack of binding as expected for the negative control, so no equation parameters are presented for DMSO.

| Parameter | Estimate | Std. Error |
| --- | --- | --- |
| c:WT+IAA | 346.04 | 0.94 |
| c:WT+2,4-D | 31.75 | 0.19 |
| c:WT+Dicamba | 24.54 | 0.32 |
| c:Mut+IAA | 54.78 | 0.28 |
| c:Mut+2,4-D | 27.16 | 0.13 |
| c:Mut+Dicamba | 20.59 | 0.16 |
| d:WT+IAA | 502.13 | 0.35 |
| d:WT+2,4-D | 260.17 | 0.48 |
| d:WT+Dicamba | 108.86 | 0.41 |
| d:Mut+IAA | 157.84 | 0.43 |
| d:Mut+2,4-D | 73.87 | 0.71 |
| d:Mut+Dicamba | 26.87 | 0.47 |
| $\propto$:WT+IAA | 145.19 | 1.86 |
| $\propto$:WT+2,4-D | 48.96 | 0.21 |
| $\propto$:WT+Dicamba | 76.08 | 1.02 |
| $\propto$:Mut+IAA | 67.70 | 0.71 |
| $\propto$:Mut+2,4-D | 21.90 | 0.59 |
| $\propto$:Mut+Dicamba | 42.55 | 5.87 |

Table S8. Results of t-tests for comparing the rate of decay in binding intensity for different degron/auxin combinations. Estimates greater than 1 indicate wildtype decay in binding is slower than mutant.

| Comparison | Estimate | Std. Error | t-value | p-value |
| --- | --- | --- | --- | --- |
| WT+IAA/Mut+IAA | 2.14 | 0.04 | 32.18 | <2.20E-16 |
| WT+2,4-D/Mut+2,4-D | 2.24 | 0.06 | 20.18 | <2.20E-16 |
| WT+Dicamba/Mut+Dicamba | 1.79 | 0.25 | 3.18 | 0.001484 |

Table S9. KASP primer set for identification of M32 allele of IAA16. The primer that binds to the wildtype has a FAM tail (green) and the primer that binds the M32 allele has a HEX tail (orange). Thermocycler conditions are 94C for 15 min; 10 cycles of 94C for 20 seconds, 61 C for 60 seconds and decreasing 0.6 C each cycle; 40 cycles of 94C for 20 seconds, 55 C for 60 seconds, quantifying FAM and HEX fluorescence at each round at 30C.

| Primer name | Sequence (5’-3’) |
| --- | --- |
| BsIAA16_intron1F_WT_KASP | GAAGGTGACCAAGTTCATGCTCTTCAGGACACAAGTTGTAGGT |
| BsIAA16_intron1F_M32_KASP | GAAGGTCGGAGTCAACGGATTGCCACACCGTATGATAGCAC |
| BsIAA16_484R_KASP | CGCTTGTGATGGCATTGCTA |

Table S10. Polymerase chain reaction primers used to amplify *BsIAA16* from cDNA libraries. Thermocycler conditions were 95 C for 5 minutes; 35 cycles of 95 C for 15 seconds, 60 C for 15 seconds, 72 C for 1 minute; 72 C for 5 minutes.

| Primer name | Sequence (5’-3’) |
| --- | --- |
| BsIAA16_1F | ATGTTGAGTAACGAGAGAGAC |
| BsIAA16_843R | TCAGCTTCTGTTCTTGCACT |

Table S11. Polymerase chain reaction primers used to clone *BsAUX/IAA16* into pFGC5941. Thermocycler conditions were 95 C for 5 minutes; 35 cycles of 95 C for 15 seconds, 60 C for 15 seconds, 72 C for 1 minute; 72 C for 5 minutes. Tails used for In-Fusion cloning are colored in green.

| Primer name | Sequence (5’-3’) |
| --- | --- |
| BsIAA16_clone_1F | TTACCATGGGGCGCGATGTTGAGTAACGAGAGAGACAAG |
| BsIAA16_clone_843R | GACTCACCTAGGATCTCAGCTTCTGTTCTTGCACTTC |

Table S12. Polymerase chain reaction primers used to amplify a section of pFGC5941 that contains the transgene of interest. Thermocycler conditions were 95 C for 5 minutes; 35 cycles of 95 C for 15 seconds, 60 C for 15 seconds, 72 C for 2 minutes; 72 C for 5 minutes. Positive samples should show an amplicon of ~400bp + the length of your transgene.

| Primer name | Sequence (5’-3’) |
| --- | --- |
| pFGC_F | CCAACCACGTCTTCAAAGCA |
| pFGC_R | GGCGTCTCGCATATCTCATT |

Table S13. Quantitative PCR primers to quantify expression of various *Arabidopsis thaliana* and *Bassia scoparia* genes. Thermocycler conditions for all primer sets are 95C for 3 minutes; 40 cycles of 95C for 15 seconds, 60C for 30 seconds, quantifying SYBR fluorescence after each round at 60C.

| Primer name | Sequence (5’-3’) |
| --- | --- |
| BsIAA16_qPCR_393R | GCGACGATGTTCTTCCTGAA |
| BsIAA16_qPCR_209F | GCAACCAAAACTACAGCGGA |
| BsSAUR21_qPCR_1F | ATGGCAATCCGATTTCCTTCAG |
| BsSAUR21_qPCR_84R | GATGTACCCTTTTGGAACTGCTT |
| BsActin_R | ATGAGAGAACGGCCTGAATG |
| BsActin_F | GAGCATCCTGTCTTACTGACTG |
| AtCyclophilin_qPCR_R | AATCGGCAACAACCACAGGC |
| AtCyclophilin_qPCR_F | GTCTGATAGAGATCTCACGT |
| AtGH3.3_qPCR_R | CGTCATTTGGAGGATTGGTTTG |
| AtGH3.3_qPCR_F | GAGAGCAAGGAACTCGTGTTAT |
| AtIAA19_qPCR_R | CTCCGTGAAAGCTCTCTTCTTC |
| AtIAA19_qPCR_F | AGGACTCGGGCTTGAGATAA |

Table S14. Phenotypic response of kochia from a F_3_ populations segregating for herbicide resistance. Visual injury was rated 21 days after treatment on a scale from 0-100 and survival was noted (A=alive, D=dead).

| **Sample** | **Injury percentage** | **Survival** |
| --- | --- | --- |
| 7710xM32-4-1-1-1 | 50 | A |
| 7710xM32-4-1-1-2 | 0 | A |
| 7710xM32-4-1-1-3 | 0 | A |
| 7710xM32-4-1-1-4 | 100 | D |
| 7710xM32-4-1-1-5 | 0 | A |
| 7710xM32-4-1-1-6 | 100 | D |
| 7710xM32-4-1-1-7 | 0 | A |
| 7710xM32-4-1-1-8 | 0 | A |
| 7710xM32-4-1-1-9 | 0 | A |
| 7710xM32-4-1-1-10 | 0 | A |
| 7710xM32-4-1-1-11 | 0 | A |
| 7710xM32-4-1-1-12 | 0 | A |
| 7710xM32-4-1-1-13 | 0 | A |
| 7710xM32-4-1-1-14 | 0 | A |
| 7710xM32-4-1-1-15 | 100 | D |
| 7710xM32-4-1-1-16 | 100 | D |
| 7710xM32-4-1-1-17 | 100 | D |
| 7710xM32-4-1-1-18 | 20 | A |
| 7710xM32-4-1-1-19 | 0 | A |
| 7710xM32-4-1-1-20 | 100 | D |
| 7710xM32-4-1-1-21 | 100 | D |
| 7710xM32-4-1-1-22 | 100 | D |
| 7710xM32-4-1-1-23 | 0 | A |
| 7710xM32-4-1-1-24 | 30 | A |
| 7710xM32-4-1-1-25 | 0 | A |
| 7710xM32-4-1-1-26 | 0 | A |
| 7710xM32-4-1-1-27 | 50 | A |
| 7710xM32-4-1-1-28 | 0 | A |
| 7710xM32-4-1-1-29 | 0 | A |
| 7710xM32-4-1-1-30 | 0 | A |
| 7710xM32-4-1-1-31 | 0 | A |
| 7710xM32-4-1-1-32 | 0 | A |
| 7710xM32-4-1-1-33 | 0 | A |
| 7710xM32-4-1-1-34 | 30 | A |
| 7710xM32-4-1-1-35 | 30 | A |
| 7710xM32-4-1-1-36 | 0 | A |
| 7710xM32-4-1-1-37 | 0 | A |
| 7710xM32-4-1-1-38 | 0 | A |
| 7710xM32-4-1-1-39 | 0 | A |
| 7710xM32-4-1-1-40 | 0 | A |
| 7710xM32-4-1-1-41 | 0 | A |
| 7710xM32-4-1-1-42 | 100 | D |
| 7710xM32-4-1-1-43 | 0 | A |
| 7710xM32-4-1-1-44 | 0 | A |
| 7710xM32-4-1-1-45 | 100 | D |
| 7710xM32-4-1-1-46 | 20 | A |
| 7710xM32-4-1-1-47 | 30 | A |
| 7710xM32-4-1-1-48 | 0 | A |
| 7710xM32-4-1-1-49 | 100 | D |
| 7710xM32-4-1-1-50 | 100 | D |
| 7710xM32-4-1-1-51 | 100 | D |
| 7710xM32-4-1-1-52 | 100 | D |
| 7710xM32-4-1-1-53 | 50 | A |
| 7710xM32-4-1-1-54 | 40 | A |
| 7710xM32-4-1-1-55 | 30 | A |
| 7710xM32-4-1-1-56 | 20 | A |
| 7710xM32-4-1-1-57 | 50 | A |
| 7710xM32-4-1-1-58 | 20 | A |
| 7710xM32-4-1-1-59 | 40 | A |
| 7710xM32-4-1-1-60 | 30 | A |
| 7710xM32-4-1-1-61 | 40 | A |
| 7710xM32-4-1-1-62 | 50 | A |
| 7710xM32-4-1-1-63 | 40 | A |
| 7710xM32-4-1-1-64 | 50 | A |
| 7710xM32-4-1-1-65 | 30 | A |
| 7710xM32-4-1-1-66 | 0 | A |
| 7710xM32-4-1-1-67 | 50 | A |
| 7710xM32-4-1-1-68 | 0 | A |
| 7710xM32-4-1-1-69 | 0 | A |
| 7710xM32-4-1-1-70 | 0 | A |
| 7710xM32-4-1-1-71 | 50 | A |
| 7710xM32-4-1-1-72 | 50 | A |
| 7710xM32-4-1-1-73 | 0 | A |
| 7710xM32-4-1-1-74 | 0 | A |
| 7710xM32-4-1-1-75 | 0 | A |
| 7710xM32-4-1-1-76 | 50 | A |
| 7710xM32-4-1-1-77 | 100 | D |
| 7710xM32-4-1-1-78 | 0 | A |
| 7710xM32-4-1-1-79 | 30 | A |
| 7710xM32-4-1-1-80 | 0 | A |
| 7710xM32-4-1-1-81 | 100 | D |
| 7710xM32-4-1-1-82 | 100 | D |
| 7710xM32-4-1-1-83 | 50 | A |
| 7710xM32-4-1-1-84 | 30 | A |
| 7710xM32-4-1-1-85 | 100 | D |
| 7710xM32-4-1-1-86 | 100 | D |
| 7710xM32-4-1-1-87 | 50 | A |
| 7710xM32-4-1-1-88 | 100 | D |
| 7710xM32-4-1-1-89 | 100 | D |
| 7710xM32-4-1-1-90 | 100 | D |
| 7710xM32-4-1-1-91 | 100 | D |
| 7710xM32-4-1-1-92 | 100 | D |
| 7710xM32-4-1-1-93 | 100 | D |
| 7710xM32-4-1-1-94 | 100 | D |
| 7710xM32-4-1-1-95 | 30 | A |
| 7710xM32-4-1-1-96 | 100 | D |
| 7710xM32-4-1-1-97 | 100 | D |
| 7710xM32-4-1-1-98 | 0 | A |
| 7710xM32-4-1-1-99 | 100 | D |
| 7710xM32-4-1-1-100 | 60 | A |
| 7710xM32-4-1-1-101 | 0 | A |
| 7710xM32-4-1-1-102 | 0 | A |
| 7710xM32-4-1-1-103 | 0 | A |
| 7710xM32-4-1-1-104 | 0 | A |
| 7710xM32-4-1-1-105 | 100 | D |
| 7710xM32-4-1-1-106 | 50 | A |
| 7710xM32-4-1-1-107 | 100 | D |
| 7710xM32-4-1-1-108 | 0 | A |
| 7710xM32-4-1-1-109 | 100 | D |
| 7710xM32-4-1-1-110 | 100 | D |
| 7710xM32-4-1-1-111 | 0 | A |
| 7710xM32-4-1-1-112 | 100 | D |
| 7710xM32-4-1-1-113 | 30 | A |
| 7710xM32-4-1-1-114 | 100 | D |
| 7710xM32-4-1-1-115 | 0 | A |
| 7710xM32-4-1-1-116 |  | No data |
| 7710xM32-4-1-1-117 | 20 | A |
| 7710xM32-4-1-1-118 | 100 | D |
| 7710xM32-4-1-1-119 | 30 | A |
| 7710xM32-4-1-1-120 | 20 | A |
| 7710xM32-4-1-1-121 | 50 | A |
| 7710xM32-4-1-1-122 | 0 | A |
| 7710xM32-4-1-1-123 | 100 | D |
| 7710xM32-4-1-1-124 | 30 | A |
| 7710xM32-4-1-1-125 | 100 | D |
| 7710xM32-4-1-1-126 | 100 | D |
| 7710xM32-4-1-1-127 | 50 | A |
| 7710xM32-4-1-1-128 | 30 | A |
| 7710xM32-4-1-1-129 | 20 | A |
| 7710xM32-4-1-1-130 | 20 | A |
| 7710xM32-4-1-1-131 | 20 | A |
| 7710xM32-4-1-1-132 | 100 | D |
| 7710xM32-4-1-1-133 | 20 | A |
| 7710xM32-4-1-1-134 | 20 | A |
| 7710xM32-4-1-1-135 | 20 | A |
| 7710xM32-4-1-1-136 | 20 | A |
| 7710xM32-4-1-1-137 | 100 | D |
| 7710xM32-4-1-1-138 | 20 | A |
| 7710xM32-4-1-1-139 | 100 | D |
| 7710xM32-4-1-1-140 | 100 | D |
| 7710xM32-4-1-1-141 | 20 | A |
| 7710xM32-4-1-1-142 | 30 | A |
| 7710xM32-4-1-1-143 |  | No data |
| 7710xM32-4-1-1-144 | 100 | D |
| 7710xM32-4-1-1-145 | 20 | A |
| 7710xM32-4-1-1-146 | 20 | A |
| 7710xM32-4-1-1-147 | 100 | D |
| 7710xM32-4-1-1-148 | 20 | A |
| 7710xM32-4-1-1-149 | 30 | A |
| 7710xM32-4-1-1-150 | 100 | D |
| 7710xM32-4-1-1-151 | 100 | D |
| 7710xM32-4-1-1-152 | 20 | A |
| 7710xM32-4-1-1-153 | 100 | D |
| 7710xM32-4-1-1-154 | 100 | D |
| 7710xM32-4-1-1-155 | 100 | D |
| 7710xM32-4-1-1-156 | 20 | A |
| 7710xM32-4-1-1-157 | 100 | D |
| 7710xM32-4-1-1-158 | 100 | D |
| 7710xM32-4-1-1-159 | 20 | A |
| 7710xM32-4-1-1-160 | 20 | A |
| 7710xM32-4-1-1-161 | 50 | A |
| 7710xM32-4-1-1-162 | 50 | A |
| 7710xM32-4-1-1-163 | 0 | A |
| 7710xM32-4-1-1-164 | 50 | A |
| 7710xM32-4-1-1-165 | 50 | A |
| 7710xM32-4-1-1-166 | 50 | A |
| 7710xM32-4-1-1-167 | 100 | D |
| 7710xM32-4-1-1-168 | 50 | A |
| 7710xM32-4-1-1-169 | 50 | A |
| 7710xM32-4-1-1-170 | 100 | D |
| 7710xM32-4-1-1-171 | 100 | D |
| 7710xM32-4-1-1-172 | 50 | A |
| 7710xM32-4-1-1-173 | 100 | D |
| 7710xM32-4-1-1-174 | 30 | A |
| 7710xM32-4-1-1-175 | 50 | A |
| 7710xM32-4-1-1-176 | 50 | A |
| 7710xM32-4-1-1-177 | 100 | D |
| 7710xM32-4-1-1-178 | 50 | A |
| 7710xM32-4-1-1-179 | 100 | D |
| 7710xM32-4-1-1-180 | 50 | A |
| 7710xM32-4-1-1-181 | 0 | A |
| 7710xM32-4-1-1-182 | 100 | D |
| 7710xM32-4-1-1-183 | 30 | A |
| 7710xM32-4-1-1-184 | 50 | A |
| 7710xM32-4-1-1-185 | 20 | A |
| 7710xM32-4-1-1-186 | 0 | A |
| 7710xM32-4-1-1-187 |  | No data |
| 7710xM32-4-1-1-188 | 50 | A |
| 7710xM32-4-1-1-189 | 20 | A |
| 7710xM32-4-1-1-190 | 0 | A |
| 7710xM32-4-1-1-191 | 100 | D |
| 7710xM32-4-1-1-192 | 100 | D |
| 7710xM32-4-1-1-193 | 0 | A |
| 7710xM32-4-1-1-194 | 50 | A |
| 7710xM32-4-1-1-195 | 100 | D |
| 7710xM32-4-1-1-196 | 20 | A |
| 7710xM32-4-1-1-197 | 30 | A |
| 7710xM32-4-1-1-198 | 0 | A |
| 7710xM32-4-1-1-199 | 30 | A |
| 7710xM32-4-1-1-200 | 0 | A |
| 7710xM32-4-1-1-201 | 0 | A |
| 7710xM32-4-1-1-202 | 30 | A |
| 7710xM32-4-1-1-203 | 0 | A |
| 7710xM32-4-1-1-204 | 30 | A |
| 7710xM32-4-1-1-205 | 30 | A |
| 7710xM32-4-1-1-206 | 30 | A |
| 7710xM32-4-1-1-207 | 30 | A |
| 7710xM32-4-1-1-208 | 100 | D |
| 7710xM32-4-1-1-209 | 30 | A |
| 7710xM32-4-1-1-210 | 30 | A |
| 7710xM32-4-1-1-211 | 30 | A |
| 7710xM32-4-1-1-212 | 30 | A |
| 7710xM32-4-1-1-213 | 0 | A |
| 7710xM32-4-1-1-214 | 30 | A |
| 7710xM32-4-1-1-215 | 0 | A |
| 7710xM32-4-1-1-216 | 30 | A |
| 7710xM32-4-1-1-217 | 30 | A |
| 7710xM32-4-1-1-218 | 0 | A |
| 7710xM32-4-1-1-219 | 0 | A |
| 7710xM32-4-1-1-220 | 30 | A |
| 7710xM32-4-1-1-221 | 30 | A |
| 7710xM32-4-1-1-222 | 30 | A |
| 7710xM32-4-1-1-223 | 30 | A |
| 7710xM32-4-1-1-224 | 30 | A |
| 7710xM32-4-1-1-225 | 100 | D |
| 7710xM32-4-1-1-226 | 100 | D |
| 7710xM32-4-1-1-227 | 50 | A |
| 7710xM32-4-1-1-228 | 30 | A |
| 7710xM32-4-1-1-229 | 100 | D |
| 7710xM32-4-1-1-230 | 100 | D |
| 7710xM32-4-1-1-231 | 0 | A |
| 7710xM32-4-1-1-232 | 100 | D |
| 7710xM32-4-1-1-233 | 100 | D |
| 7710xM32-4-1-1-234 | 0 | A |
| 7710xM32-4-1-1-235 | 30 | A |
| 7710xM32-4-1-1-236 | 100 | D |
| 7710xM32-4-1-1-237 | 0 | A |
| 7710xM32-4-1-1-238 | 0 | A |
| 7710xM32-4-1-1-239 | 30 | A |
| 7710xM32-4-1-1-240 | 30 | A |
| 7710xM32-4-1-1-241 | 0 | A |
| 7710xM32-4-1-1-242 | 100 | D |
| 7710xM32-4-1-1-243 | 100 | D |
| 7710xM32-4-1-1-244 | 20 | A |
| 7710xM32-4-1-1-245 | 100 | D |
| 7710xM32-4-1-1-246 | 0 | A |
| 7710xM32-4-1-1-247 | 0 | A |
| 7710xM32-4-1-1-248 | 20 | A |
| 7710xM32-4-1-1-249 | 0 | A |
| 7710xM32-4-1-1-250 | 0 | A |
| 7710xM32-4-1-1-251 | 0 | A |
| 7710xM32-4-1-1-252 | 20 | A |
| 7710xM32-4-1-1-253 | 100 | D |
| 7710xM32-4-1-1-254 | 0 | A |
| 7710xM32-4-1-1-255 | 20 | A |
| 7710xM32-4-1-1-256 | 100 | D |
| 7710xM32-4-1-1-257 | 0 | A |
| 7710xM32-4-1-1-258 | 0 | A |
| 7710xM32-4-1-1-259 | 20 | A |
| 7710xM32-4-1-1-260 | 100 | D |
| 7710xM32-4-1-1-261 | 40 | A |
| 7710xM32-4-1-1-262 | 30 | A |
| 7710xM32-4-1-1-263 | 100 | D |
| 7710xM32-4-1-1-264 | 40 | A |
| 7710xM32-4-1-1-265 | 100 | D |
| 7710xM32-4-1-1-266 | 30 | A |
| 7710xM32-4-1-1-267 | 100 | D |
| 7710xM32-4-1-1-268 | 20 | A |
| 7710xM32-4-1-1-269 | 100 | D |
| 7710xM32-4-1-1-270 | 30 | A |
| 7710xM32-4-1-1-271 | 30 | A |
| 7710xM32-4-1-1-272 | 100 | D |
| 7710xM32-4-1-1-273 | 40 | A |
| 7710xM32-4-1-1-274 | 100 | D |
| 7710xM32-4-1-1-275 | 30 | A |
| 7710xM32-4-1-1-276 | 20 | A |
| 7710xM32-4-1-1-277 | 30 | A |
| 7710xM32-4-1-1-278 | 30 | A |
| 7710xM32-4-1-1-279 | 100 | D |
| 7710xM32-4-1-1-280 | 100 | D |
| 7710xM32-4-1-1-281 | 30 | A |
| 7710xM32-4-1-1-282 | 30 | A |
| 7710xM32-4-1-1-283 | 30 | A |
| 7710xM32-4-1-1-284 | 30 | A |
| 7710xM32-4-1-1-285 | 100 | D |
| 7710xM32-4-1-1-286 | 30 | A |
| 7710xM32-4-1-1-287 | 30 | A |
| 7710xM32-4-1-1-288 | 0 | A |
| 7710-M32-4-5-10-1 | 100 | D |
| 7710-M32-4-5-10-2 | 100 | D |
| 7710-M32-4-5-10-3 | 70 | A |
| 7710-M32-4-5-10-4 | 100 | D |
| 7710-M32-4-5-10-5 | 30 | A |
| 7710-M32-4-5-10-6 | 70 | A |
| 7710-M32-4-5-10-7 |  | No data |
| 7710-M32-4-5-10-8 | 100 | D |
| 7710-M32-4-5-10-9 | 30 | A |
| 7710-M32-4-5-10-10 | 100 | D |
| 7710-M32-4-5-10-11 | 20 | A |
| 7710-M32-4-5-10-12 | 70 | A |
| 7710-M32-4-5-10-13 | 100 | D |
| 7710-M32-4-5-10-14 | 20 | A |
| 7710-M32-4-5-10-15 | 20 | A |
| 7710-M32-4-5-10-16 | 20 | A |
| 7710-M32-4-5-10-17 | 100 | D |
| 7710-M32-4-5-10-18 | 20 | A |
| 7710-M32-4-5-10-19 | 80 | A |
| 7710-M32-4-5-10-20 | 100 | D |
| 7710-M32-4-5-10-21 | 100 | D |
| 7710-M32-4-5-10-22 | 20 | A |
| 7710-M32-4-5-10-23 |  | No data |
| 7710-M32-4-5-10-24 | 20 | A |
| 7710-M32-4-5-10-25 | 20 | A |
| 7710-M32-4-5-10-26 | 70 | A |
| 7710-M32-4-5-10-27 | 30 | A |
| 7710-M32-4-5-10-28 | 20 | A |
| 7710-M32-4-5-10-29 | 20 | A |
| 7710-M32-4-5-10-30 | 20 | A |
| 7710-M32-4-5-10-31 | 20 | A |
| 7710-M32-4-5-10-32 |  | No data |
| 7710-M32-4-5-10-33 | 100 | D |
| 7710-M32-4-5-10-34 | 20 | A |
| 7710-M32-4-5-10-35 | 100 | D |
| 7710-M32-4-5-10-36 | 100 | D |
| 7710-M32-4-5-10-37 | 100 | D |
| 7710-M32-4-5-10-38 | 80 | A |
| 7710-M32-4-5-10-39 | 100 | D |
| 7710-M32-4-5-10-40 |  | No data |
| 7710-M32-4-5-10-41 | 100 | D |
| 7710-M32-4-5-10-42 | 30 | A |
| 7710-M32-4-5-10-43 | 100 | D |
| 7710-M32-4-5-10-44 |  | No data |
| 7710-M32-4-5-10-45 | 100 | D |
| 7710-M32-4-5-10-46 | 100 | D |
| 7710-M32-4-5-10-47 | 20 | A |
| 7710-M32-4-5-10-48 | 80 | A |
| 7710-M32-4-5-10-49 | 30 | A |
| 7710-M32-4-5-10-50 | 30 | A |
| 7710-M32-4-5-10-51 | 100 | D |
| 7710-M32-4-5-10-52 | 30 | A |
| 7710-M32-4-5-10-53 | 30 | A |
| 7710-M32-4-5-10-54 | 100 | D |
| 7710-M32-4-5-10-55 | 100 | D |
| 7710-M32-4-5-10-56 | 100 | D |
| 7710-M32-4-5-10-57 |  | No data |
| 7710-M32-4-5-10-58 | 50 | A |
| 7710-M32-4-5-10-59 | 100 | D |
| 7710-M32-4-5-10-60 | 100 | D |
| 7710-M32-4-5-10-61 | 80 | A |
| 7710-M32-4-5-10-62 | 80 | A |
| 7710-M32-4-5-10-63 | 80 | A |
| 7710-M32-4-5-10-64 | 100 | D |
| 7710-M32-4-5-10-65 | 0 | A |
| 7710-M32-4-5-10-66 | 100 | D |
| 7710-M32-4-5-10-67 | 0 | A |
| 7710-M32-4-5-10-68 | 100 | D |
| 7710-M32-4-5-10-69 | 0 | A |
| 7710-M32-4-5-10-70 | 0 | A |
| 7710-M32-4-5-10-71 | 0 | A |
| 7710-M32-4-5-10-72 | 0 | A |
| 7710-M32-4-5-10-73 | 0 | A |
| 7710-M32-4-5-10-74 | 100 | D |
| 7710-M32-4-5-10-75 | 100 | D |
| 7710-M32-4-5-10-76 | 0 | A |
| 7710-M32-4-5-10-77 | 0 | A |
| 7710-M32-4-5-10-78 | 100 | D |
| 7710-M32-4-5-10-79 | 0 | A |
| 7710-M32-4-5-10-80 | 0 | A |
| 7710-M32-4-5-10-81 | 0 | A |
| 7710-M32-4-5-10-82 | 0 | A |
| 7710-M32-4-5-10-83 | 0 | A |
| 7710-M32-4-5-10-84 | 0 | A |
| 7710-M32-4-5-10-85 | 100 | D |
| 7710-M32-4-5-10-86 | 20 | A |
| 7710-M32-4-5-10-87 | 0 | A |
| 7710-M32-4-5-10-88 | 100 | D |
| 7710-M32-4-5-10-89 | 100 | D |
| 7710-M32-4-5-10-90 | 100 | D |
| 7710-M32-4-5-10-91 |  | No data |
| 7710-M32-4-5-10-92 | 0 | A |
| 7710-M32-4-5-10-93 | 0 | A |
| 7710-M32-4-5-10-94 | 30 | A |
| 7710-M32-4-5-10-95 | 100 | D |
| 7710-M32-4-5-10-96 | 100 | D |
| 7710-M32-4-5-10-97 | 20 | A |
| 7710-M32-4-5-10-98 | 20 | A |
| 7710-M32-4-5-10-99 | 100 | D |
| 7710-M32-4-5-10-100 | 20 | A |
| 7710-M32-4-5-10-101 | 20 | A |
| 7710-M32-4-5-10-102 | 20 | A |
| 7710-M32-4-5-10-103 | 20 | A |
| 7710-M32-4-5-10-104 | 20 | A |
| 7710-M32-4-5-10-105 | 20 | A |
| 7710-M32-4-5-10-106 | 20 | A |
| 7710-M32-4-5-10-107 | 100 | D |
| 7710-M32-4-5-10-108 | 20 | A |
| 7710-M32-4-5-10-109 | 80 | A |
| 7710-M32-4-5-10-110 | 20 | A |
| 7710-M32-4-5-10-111 | 20 | A |
| 7710-M32-4-5-10-112 | 20 | A |
| 7710-M32-4-5-10-113 | 20 | A |
| 7710-M32-4-5-10-114 | 80 | A |
| 7710-M32-4-5-10-115 | 100 | D |
| 7710-M32-4-5-10-116 | 20 | A |
| 7710-M32-4-5-10-117 | 20 | A |
| 7710-M32-4-5-10-118 | 20 | A |
| 7710-M32-4-5-10-119 | 20 | A |
| 7710-M32-4-5-10-120 | 20 | A |
| 7710-M32-4-5-10-121 | 100 | D |
| 7710-M32-4-5-10-122 | 20 | A |
| 7710-M32-4-5-10-123 | 100 | D |
| 7710-M32-4-5-10-124 | 20 | A |
| 7710-M32-4-5-10-125 | 20 | A |
| 7710-M32-4-5-10-126 | 20 | A |
| 7710-M32-4-5-10-127 | 100 | D |
| 7710-M32-4-5-10-128 | 100 | D |
| 7710-M32-4-5-10-129 | 100 | D |
| 7710-M32-4-5-10-130 | 0 | A |
| 7710-M32-4-5-10-131 | 0 | A |
| 7710-M32-4-5-10-132 | 80 | A |
| 7710-M32-4-5-10-133 | 20 | A |
| 7710-M32-4-5-10-134 |  | No data |
| 7710-M32-4-5-10-135 | 0 | A |
| 7710-M32-4-5-10-136 | 0 | A |
| 7710-M32-4-5-10-137 | 100 | D |
| 7710-M32-4-5-10-138 |  | No data |
| 7710-M32-4-5-10-139 | 100 | D |
| 7710-M32-4-5-10-140 | 100 | D |
| 7710-M32-4-5-10-141 |  | No data |
| 7710-M32-4-5-10-142 |  | No data |
| 7710-M32-4-5-10-143 |  | No data |
| 7710-M32-4-5-10-144 |  | No data |
| 7710-M32-4-5-10-145 |  | No data |
| 7710-M32-4-5-10-146 | 100 | D |
| 7710-M32-4-5-10-147 |  | No data |
| 7710-M32-4-5-10-148 | 80 | A |
| 7710-M32-4-5-10-149 |  | No data |
| 7710-M32-4-5-10-150 | 100 | D |
| 7710-M32-4-5-10-151 |  | No data |
| 7710-M32-4-5-10-152 | 80 | A |
| 7710-M32-4-5-10-153 |  | No data |
| 7710-M32-4-5-10-154 |  | No data |
| 7710-M32-4-5-10-155 | 80 | A |
| 7710-M32-4-5-10-156 |  | No data |
| 7710-M32-4-5-10-157 | 100 | D |
| 7710-M32-4-5-10-158 | 80 | A |
| 7710-M32-4-5-10-159 | 100 | D |
| 7710-M32-4-5-10-160 | 30 | A |
| 7710-M32-4-5-10-161 | 100 | D |
| 7710-M32-4-5-10-162 | 70 | A |
| 7710-M32-4-5-10-163 | 100 | D |
| 7710-M32-4-5-10-164 | 100 | D |
| 7710-M32-4-5-10-165 | 70 | A |
| 7710-M32-4-5-10-166 | 70 | A |
| 7710-M32-4-5-10-167 | 100 | D |
| 7710-M32-4-5-10-168 | 100 | D |
| 7710-M32-4-5-10-169 | 70 | A |
| 7710-M32-4-5-10-170 | 100 | D |
| 7710-M32-4-5-10-171 |  | No data |
| 7710-M32-4-5-10-172 | 70 | A |
| 7710-M32-4-5-10-173 | 30 | A |
| 7710-M32-4-5-10-174 | 70 | A |
| 7710-M32-4-5-10-175 | 30 | A |
| 7710-M32-4-5-10-176 | 30 | A |
| 7710-M32-4-5-10-177 | 30 | A |
| 7710-M32-4-5-10-178 |  | No data |
| 7710-M32-4-5-10-179 | 100 | D |
| 7710-M32-4-5-10-180 | 30 | A |
| 7710-M32-4-5-10-181 | 100 | D |
| 7710-M32-4-5-10-182 | 100 | D |
| 7710-M32-4-5-10-183 | 30 | A |
| 7710-M32-4-5-10-184 | 100 | D |
| 7710-M32-4-5-10-185 | 30 | A |
| 7710-M32-4-5-10-186 |  | No data |
| 7710-M32-4-5-10-187 |  | No data |
| 7710-M32-4-5-10-188 | 30 | A |
| 7710-M32-4-5-10-189 | 100 | D |
| 7710-M32-4-5-10-190 | 30 | A |
| 7710-M32-4-5-10-191 | 20 | A |
| 7710-M32-4-5-10-192 | 70 | A |
| 7710-M32-4-5-10-193 | 100 | D |
| 7710-M32-4-5-10-194 | 80 | A |
| 7710-M32-4-5-10-195 |  | No data |
| 7710-M32-4-5-10-196 |  | No data |
| 7710-M32-4-5-10-197 | 30 | A |
| 7710-M32-4-5-10-198 |  | No data |
| 7710-M32-4-5-10-199 |  | No data |
| 7710-M32-4-5-10-200 |  | No data |
| 7710-M32-4-5-10-201 | 30 | A |
| 7710-M32-4-5-10-202 | 80 | A |
| 7710-M32-4-5-10-203 |  | No data |
| 7710-M32-4-5-10-204 | 100 | D |
| 7710-M32-4-5-10-205 |  | No data |
| 7710-M32-4-5-10-206 | 100 | D |
| 7710-M32-4-5-10-207 | 30 | A |
| 7710-M32-4-5-10-208 | 100 | D |
| 7710-M32-4-5-10-209 |  | No data |
| 7710-M32-4-5-10-210 | 30 | A |
| 7710-M32-4-5-10-211 | 30 | A |
| 7710-M32-4-5-10-212 | 30 | A |
| 7710-M32-4-5-10-213 | 100 | D |
| 7710-M32-4-5-10-214 | 30 | A |
| 7710-M32-4-5-10-215 | 100 | D |
| 7710-M32-4-5-10-216 | 30 | A |
| 7710-M32-4-5-10-217 | 30 | A |
| 7710-M32-4-5-10-218 | 30 | A |
| 7710-M32-4-5-10-219 | 30 | A |
| 7710-M32-4-5-10-220 | 100 | D |
| 7710-M32-4-5-10-221 | 100 | D |
| 7710-M32-4-5-10-222 | 30 | A |
| 7710-M32-4-5-10-223 | 100 | D |
| 7710-M32-4-5-10-224 | 80 | A |
| 7710-M32-4-5-10-225 | 20 | A |
| 7710-M32-4-5-10-226 | 100 | D |
| 7710-M32-4-5-10-227 | 100 | D |
| 7710-M32-4-5-10-228 |  | No data |
| 7710-M32-4-5-10-229 | 20 | A |
| 7710-M32-4-5-10-230 |  | No data |
| 7710-M32-4-5-10-231 | 20 | A |
| 7710-M32-4-5-10-232 | 100 | D |
| 7710-M32-4-5-10-233 |  | No data |
| 7710-M32-4-5-10-234 | 0 | A |
| 7710-M32-4-5-10-235 |  | No data |
| 7710-M32-4-5-10-236 |  | No data |
| 7710-M32-4-5-10-237 | 100 | D |
| 7710-M32-4-5-10-238 | 0 | A |
| 7710-M32-4-5-10-239 | 0 | A |
| 7710-M32-4-5-10-240 | 80 | A |
| 7710-M32-4-5-10-241 | 30 | A |
| 7710-M32-4-5-10-242 |  | No data |
| 7710-M32-4-5-10-243 | 0 | A |
| 7710-M32-4-5-10-244 | 100 | D |
| 7710-M32-4-5-10-245 |  | No data |
| 7710-M32-4-5-10-246 |  | No data |
| 7710-M32-4-5-10-247 | 0 | A |
| 7710-M32-4-5-10-248 | 100 | D |
| 7710-M32-4-5-10-249 | 100 | D |
| 7710-M32-4-5-10-250 | 0 | A |
| 7710-M32-4-5-10-251 | 30 | A |
| 7710-M32-4-5-10-252 | 0 | A |
| 7710-M32-4-5-10-253 | 0 | A |
| 7710-M32-4-5-10-254 | 0 | A |
| 7710-M32-4-5-10-255 | 100 | D |
| 7710-M32-4-5-10-256 | 0 | A |
| 7710-M32-4-5-10-257 | 0 | A |
| 7710-M32-4-5-10-258 | 100 | D |
| 7710-M32-4-5-10-259 | 100 | D |
| 7710-M32-4-5-10-260 | 100 | D |
| 7710-M32-4-5-10-261 | 100 | D |
| 7710-M32-4-5-10-262 | 100 | D |
| 7710-M32-4-5-10-263 | 0 | A |
| 7710-M32-4-5-10-264 | 100 | D |
| 7710-M32-4-5-10-265 | 80 | A |
| 7710-M32-4-5-10-266 | 80 | A |
| 7710-M32-4-5-10-267 | 0 | A |
| 7710-M32-4-5-10-268 |  | No data |
| 7710-M32-4-5-10-269 | 100 | D |
| 7710-M32-4-5-10-270 | 0 | A |
| 7710-M32-4-5-10-271 | 100 | D |
| 7710-M32-4-5-10-272 | 20 | A |
| 7710-M32-4-5-10-273 | 0 | A |
| 7710-M32-4-5-10-274 | 80 | A |
| 7710-M32-4-5-10-275 | 100 | D |
| 7710-M32-4-5-10-276 | 100 | D |
| 7710-M32-4-5-10-277 | 30 | A |
| 7710-M32-4-5-10-278 | 100 | D |
| 7710-M32-4-5-10-279 | 80 | A |
| 7710-M32-4-5-10-280 | 100 | D |
| 7710-M32-4-5-10-281 | 100 | D |
| 7710-M32-4-5-10-282 | 100 | D |
| 7710-M32-4-5-10-283 | 100 | D |
| 7710-M32-4-5-10-284 | 20 | A |
| 7710-M32-4-5-10-285 | 0 | A |
| 7710-M32-4-5-10-286 | 0 | A |
| 7710-M32-4-5-10-287 |  | No data |
| 7710-M32-4-5-10-288 | 100 | D |
| 7710-M32-4-5-10-289 | 80 | A |
| 7710-M32-4-5-10-290 |  | No data |
| 7710-M32-4-5-10-291 | 100 | D |
| 7710-M32-4-5-10-292 | 30 | A |
| 7710-M32-4-5-10-293 | 30 | A |
| 7710-M32-4-5-10-294 | 30 | A |
| 7710-M32-4-5-10-295 | 100 | D |
| 7710-M32-4-5-10-296 | 100 | D |
| 7710-M32-4-5-10-297 | 30 | A |
| 7710-M32-4-5-10-298 | 30 | A |
| 7710-M32-4-5-10-299 | 30 | A |
| 7710-M32-4-5-10-300 | 30 | A |
| 7710-M32-4-5-10-301 | 30 | A |
| 7710-M32-4-5-10-302 | 30 | A |
| 7710-M32-4-5-10-303 | 30 | A |
| 7710-M32-4-5-10-304 | 100 | D |
| 7710-M32-4-5-10-305 | 100 | D |
| 7710-M32-4-5-10-306 | 30 | A |
| 7710-M32-4-5-10-307 | 30 | A |
| 7710-M32-4-5-10-308 | 100 | D |
| 7710-M32-4-5-10-309 | 100 | D |
| 7710-M32-4-5-10-310 | 30 | A |
| 7710-M32-4-5-10-311 | 30 | A |
| 7710-M32-4-5-10-312 | 30 | A |
| 7710-M32-4-5-10-313 | 30 | A |
| 7710-M32-4-5-10-314 |  | No data |
| 7710-M32-4-5-10-315 | 30 | A |
| 7710-M32-4-5-10-316 | 30 | A |
| 7710-M32-4-5-10-317 | 100 | D |
| 7710-M32-4-5-10-318 | 100 | D |
| 7710-M32-4-5-10-319 | 30 | A |
| 7710-M32-4-5-10-320 | 30 | A |
| 7710-M32-4-5-10-321 | 100 | D |
| 7710-M32-4-5-10-322 | 80 | A |
| 7710-M32-4-5-10-323 | 100 | D |
| 7710-M32-4-5-10-324 |  | No data |
| 7710-M32-4-5-10-325 | 0 | A |
| 7710-M32-4-5-10-326 | 100 | D |
| 7710-M32-4-5-10-327 | 100 | D |
| 7710-M32-4-5-10-328 | 100 | D |
| 7710-M32-4-5-10-329 | 100 | D |
| 7710-M32-4-5-10-330 | 20 | A |
| 7710-M32-4-5-10-331 |  | No data |
| 7710-M32-4-5-10-332 | 80 | A |
| 7710-M32-4-5-10-333 | 80 | A |
| 7710-M32-4-5-10-334 | 0 | A |
| 7710-M32-4-5-10-335 | 0 | A |
| 7710-M32-4-5-10-336 | 30 | A |
| 7710-M32-4-5-10-337 | 30 | A |
| 7710-M32-4-5-10-338 | 100 | D |
| 7710-M32-4-5-10-339 | 100 | D |
| 7710-M32-4-5-10-340 | 0 | A |
| 7710-M32-4-5-10-341 | 0 | A |
| 7710-M32-4-5-10-342 | 0 | A |
| 7710-M32-4-5-10-343 | 0 | A |
| 7710-M32-4-5-10-344 | 0 | A |
| 7710-M32-4-5-10-345 | 0 | A |
| 7710-M32-4-5-10-346 | 0 | A |
| 7710-M32-4-5-10-347 | 100 | D |
| 7710-M32-4-5-10-348 | 0 | A |
| 7710-M32-4-5-10-349 | 80 | A |
| 7710-M32-4-5-10-350 | 20 | A |
| 7710-M32-4-5-10-351 | 0 | A |
| 7710-M32-4-5-10-352 | 80 | A |
